# The cotton MYB108 forms a positive feedback regulation loop with CML11 and participates in the defense response against *Verticillium dahliae* infection

**DOI:** 10.1093/jxb/erw016

**Published:** 2016-02-11

**Authors:** Huan-Qing Cheng, Li-Bo Han, Chun-Lin Yang, Xiao-Min Wu, Nai-Qin Zhong, Jia-He Wu, Fu-Xin Wang, Hai-Yun Wang, Gui-Xian Xia

**Affiliations:** ^1^Institute of Microbiology, Chinese Academy of Sciences, Beijing 100101, China; ^2^State Key Laboratory of Plant Genomics, Beijing 100101, China; ^3^University of Chinese Academy of Sciences, Beijing 100049, China

**Keywords:** Calcium, calmodulin, cotton, MYB, plant defense, *Verticillium dahliae*.

## Abstract

Accumulating evidence indicates that plant MYB transcription factors participate in defense against pathogen attack, but their regulatory targets and related signaling processes remain largely unknown. Here, we identified a defense-related *MYB* gene (*GhMYB108*) from upland cotton (*Gossypium hirsutum*) and characterized its functional mechanism. Expression of *GhMYB108* in cotton plants was induced by *Verticillium dahliae* infection and responded to the application of defense signaling molecules, including salicylic acid, jasmonic acid, and ethylene. Knockdown of *GhMYB108* expression led to increased susceptibility of cotton plants to *V. dahliae*, while ecotopic overexpression of *GhMYB108* in *Arabidopsis thaliana* conferred enhanced tolerance to the pathogen. Further analysis demonstrated that GhMYB108 interacted with the calmodulin-like protein GhCML11, and the two proteins form a positive feedback loop to enhance the transcription of *GhCML11* in a calcium-dependent manner. *Verticillium dahliae* infection stimulated Ca^2+^ influx into the cytosol in cotton root cells, but this response was disrupted in both *GhCML11*-silenced plants and *GhMYB108*-silenced plants in which expression of several calcium signaling-related genes was down-regulated. Taken together, these results indicate that *GhMYB108* acts as a positive regulator in defense against *V. dahliae* infection by interacting with GhCML11. Furthermore, the data also revealed the important roles and synergetic regulation of MYB transcription factor, Ca^2+^, and calmodulin in plant immune responses.

## Highlight

Cotton MYB108 interacts with CML11 and acts as a positive regulator in defense against *V. dahliae* infection.

## Introduction

Increasing numbers of transcription factors (TFs), including members of the WRKY, NAC, bHLH, bZIP, ERF/AP2, and MYB families, have been reported to play crucial roles in plant immunity (Singh *et al.*, 2002; Buscaill and Rivas, 2014). In the defense against pathogen attack, TFs modulate the transcription of defense-related genes, and they often execute such functions through interaction with other regulators (Tsuda and Somssich, 2015). MYB proteins represent one of the largest TF families and have a characteristic MYB domain consisting of 52 amino acid repeats that form helix–turn–helix structures required for binding to *cis*-elements in the promoters of target genes (Dubos *et al.*, 2010). Based on the numbers of adjacent repeats, MYB proteins are divided into four classes: R1-MYB, R2R3-MYB, 3R-MYB, and 4R-MYB (Dubos *et al.*, 2010). MYB proteins play important roles in plant development and responses, as shown for various species such as Arabidopsis (*Arabidopsis thaliana*), tobacco (*Nicotiana tabacum*), rice (*Oryza sativa*), and cotton (*Gossypium hirsutum*), and the molecular mechanisms by which these MYBs fulfill their functions are very well established (Lippold *et al.*, 2009; Liu *et al.*, 2009; Zhang *et al.*, 2010; Walford *et al.*, 2011; Yang *et al.*, 2012; Lee *et al.*, 2015). Several MYBs have been reported to function in defense against pathogens, including AtMYB30, AtBOS1 (AtMYB108), and TaPIMP1 (Vailleau *et al.*, 2002; Mengiste *et al.*, 2003; Zhang *et al.*, 2012), yet the regulatory mechanisms and signaling processes mediated by MYB proteins in defense responses remain largely unknown.

Ca^2+^ is an important second messenger for the transduction of signals regulating plant development and the response to environmental cues (Hepler, 2005; Sarwat *et al.*, 2013). Influx of Ca^2+^ into the cytosol is an important early event in pathogen attack (Lecourieux *et al.*, 2006). The major Ca^2+^ sensors include calmodulin (CaM) and CaM-like proteins, which localize in various cellular compartments such as the cytoplasm, apoplast, nucleus, and peroxisome (Yang and Poovaiah, 2003). CaMs regulate a number of downstream targets involved in diverse plant processes (Bouché *et al.*, 2005). After pathogen challenge, expression of multiple CaM genes is induced or suppressed as part of the plant defense response (Heo *et al.*, 1999; Chiasson *et al.*, 2005). Several studies reported that CaMs regulate gene expression by interacting with TFs such as members of the WRKY and CAMTA families, in plant innate immunity responses (Park *et al.*, 2005; Galon *et al.*, 2008). These studies have begun to reveal the molecular mechanisms by which Ca^2+^/CaM and TFs co-operate to modulate defense-related transcriptional responses.

Cotton Verticillium wilt is a highly destructive vascular disease that is mainly caused by the soil-borne fungus *Verticillium dahliae*, and this disease leads to severe loss of cotton yields worldwide and threatens most cotton-producing areas (Fradin and Thomma, 2006). Although long-term efforts have been made to produce wilt-resistant cotton cultivars by traditional breeding, very few varieties of upland cotton are resistant to Verticillium wilt (Cai *et al.*, 2009). During the past years, progress has been made in exploring the molecular mechanism of the disease tolerance against *V*. *dahliae* invasion in cotton, with the ultimate aim of generating Verticillium wilt-resistant cultivars by molecular breeding. Accumulating evidence indicates that sets of *V. dahliae*-responsive genes, such as *GhNDR1*, *GhNaD1*, *GhSSN*, *GbWRKY1*, and *GhMLP28* (Gao *et al.*, 2011; Gaspar *et al.*, 2014; Li *et al.*, 2014; Sun *et al.*, 2014; Yang *et al.*, 2015), are functionally related to defense responses against *V. dahliae* infection in cotton.

In this study, we identified the *V. dahliae*-responsive gene *GhMYB108* from upland cotton. Functional characterization indicates that it participates in the defense response through interaction with the CaM-like protein GhCML11. Moreover, the two proteins form a positive feedback loop to regulate the transcription of *GhCML11*. Another interesting finding of this study is that GhCML11 proteins localize in the apoplast as well as in the nucleus and cytoplasm. Apoplastic GhCML11 may be required for Ca^2+^ influx in response to pathogen attack, and nuclear GhCML11 may act with GhMYB108 to activate the transcription of defense genes. Our results provide important insights into the significance of the synergetic interaction between a MYB transcription factor and Ca^2+^/CaM in plant immune responses.

## Materials and methods

### Plant materials and growth conditions

*Gossypium hirsutum* variety BD18, kindly provided by Professor Guiliang Jian (Institute of Plant Protection, CAAS), which is a Verticillium wilt-tolerant breeding line of upland cotton, was used in this study. Cotton plants were grown in pots at 28 °C under 16h/8h light/dark conditions.

*Nicotiana benthamiana* and *A. thaliana* (ecotype Columbia-1) plants were grown in the greenhouse under 16h/8h light/dark conditions at 23 °C and watered weekly with Murashige and Skoog nutrient solution.

### Arabidopsis transformation

The ORF of *GhMYB108* was cloned under control of the 35S promoter in the plant expression vector *pBI121*. The resulting plasmid *pBI121-GhMYB108* was introduced into the *Agrobacterium tumefaciens* strain EHA105. Transformation of Arabidopsis plants was performed using the floral-dip method (Clough and Bent, 1998).

### Pathogen cultivation and inoculation

The *V. dahliae* strain *V991* originally isolated from an infected upland cotton, which is a strong pathogenic defoliating isolate (W.W. Zhang *et al.*, 2012), was used as the pathogen. Fungal colonies were cultured on potato dextrose agar plates for 1 week at 26 °C. For *V. dahliae* infection, the roots of cotton seedlings grown under hydroponic conditions for 12 d were inoculated with a spore suspension (10^6^ spores ml^−1^), and then harvested at the indicated time for RNA extraction. To infect VIGS (virus-induced gene silencing) cotton plants, the spore suspensions were stem-inoculated into cotton plants at a position 1cm under the cotyledons with a syringe needle (Bolek *et al.*, 2005), at a dose of 3 μl per plant. For Arabidopsis infection, roots of 4-week-old plants were incubated in spore suspensions for 3min. Subsequently, plants were transplanted into fresh steam-sterilized vermiculite. The disease index was calculated according to the following formula: disease index=[(∑disease grades×number of infected plants)/(total checked plants×4)]×100. Seedlings were classified into five grades (grade 0, 1, 2, 3, and 4) based on the disease severity after *V. dahliae* infection, as described by Wang *et al.* (2004).

*Pseudomonas syringae* pv. tomato strain DC3000 was grown in King’s B medium at 28 °C. Overnight culture cells were resuspended in 10mM MgCl_2_. The cell density was adjusted to 2×10^5^ colony-forming units (cfu) ml^−1^ for inoculation, and the bacterial growth was detected 3 d after inoculation. *Botrytis cinerea* strain BO5-10 was grown on potato dextrose agar at 23 °C for 10–14 d. Spores were harvested and adjusted to a concentration of 10^5^ spores ml^−1^ with distilled water. A 6 μl aliquot of spore suspension was dropped on Arabidopsis leaves and the lesion size was measured at 3 d after inoculation.

### Hormone, CaCl_2_, and LaCl_3_ treatments

Cotton roots were treated with 0.1mM salicylic acid, 0.15mM jasmonic acid, 1mM ethylene, and different concentration of CaCl_2_. Cotton roots were treated with 300 μM LaCl_3_ before and after *V. dahliae* infection. Roots treated with sterile water were used as mock control.

### RNA extraction and qRT-PCR analysis

Total RNA was extracted using TRIzol reagent (Invitrogen) according to the manufacturer’s protocol. The quantitative real-time PCR (qRT-PCR) assay was conducted using the SYBR Green Real-Time PCR Master Mix (Toyobo, Japan) and the DNA Engine Opticon 2 Real-Time PCR Detection System (MJ Research). The cotton *Histone3* gene or Arabidopsis *EF-1α* gene was used as the internal control. The expression levels of genes were calculated by using the 2^–ΔΔCT^ or 2^–ΔCT^ method, where CT is the cycle threshold. ΔCT=CT_Target_–CT_*Histone3*/*EF-1*α_. ΔΔCT=ΔCT_sample_–ΔCT_control_. The amplification efficiency of the samples was quantified and adjusted. All reactions were conducted in triplicate. The primers used in qRT-PCR are listed in Supplementary Table S1 at *JXB* online.

### Electrophoretic mobility shift assay

The ORF of *GhMYB108* was cloned into the *pMAL-p2X* vector to produce maltose-binding protein (MBP)–GhMYB108 fusion protein. The ORF of *GhCML11* was fused to the *pGEX6P-1* vector to produce glutathione *S*-transferase (GST)–GhCML11 fusion protein. Fusion proteins were expressed in *Escherichia coli* strain BL21 and purified. EMSA was performed using biotin-labeled probes and a Pierce LightShift Chemiluminescent EMSA kit (Thermo). The binding reaction was carried out in a 20 μl reaction mixture at room temperature for 30min and then separated on a native 6% polyacrylamide gel in 0.5× Tris-borate/EDTA buffer. To test the effect of GhCML11 on the DNA binding activity of GhMYB108, the reaction mixtures were supplemented with CaCl_2_ (10 μM) or EGTA (0.5mM), or purified GST–GhCML11 fusion proteins. The labeled probes were detected based on the manufacturer’s instructions.

### Dual-luciferase reporter (DLR) assay

The TF activity of GhMYB108 was examined by DLR assay as described by Ohta *et al.* (2001). The reporter plasmid contains the *Luc* gene, controlled by the minimal TATA region of the *35S* promoter with five GAL4-binding elements upstream. The *Renilla luciferase* gene driven by the *35S* promoter was used as an internal control. To construct effector plasmids, the ORF of *GhMYB108* was introduced into the *pRT-BD* vector to generate *35S-BD-GhMYB108*. The Arabidopsis protoplasts were transfected with a mixture of 6 μg of effector plasmid, 6 μg of reporter plasmid, and 1 μg of internal control plasmid by polyethylene glycol (PEG) transformation. After 16h under dark conditions, the luciferase assay was conducted using the DLR assay system (Promega) and a GloMax 20-20 luminometer (Bio-rad).

### Subcellular localization

The ORF of *GhMYB108* was fused to *GFP* (green fluorescent protein) and the ORF of *GhCML11* was fused to *mCherry* under the control of the *35S* promoter in the expression vector *pPZP111* (Hajdukiewicz *et al.*, 1994) to generate *pPZP-GhMYB108-GFP* and *pPZP-GhCML11-mCherry* constructs, respectively. *Agrobacterium* cells (strain GV3101) containing recombinant plasmids were infiltrated into *N. benthamiana* leaves. The infiltrated plants were incubated for 40h at 23 °C under dark conditions. The cells expressing GFP proteins were stained with DAPI to indicate the nucleus. The signals were visualized with a confocal microscope (Leica TCS SP8, Germany).

For visualization of GhCML11 in onion epidermal cells, 2 μg of plasmid DNA was used to coat gold particles, and the plasmid harboring *GFP* alone was used as the control. The inner layer of the onion epidermis was bombarded by a gene gun (PDS-1000/He, Bio-Rad). For plasmolysis experiment, cells were treated with 20% sucrose. Signals were visualized by confocal microscopy.

### Virus-induced gene silencing

*pTRV1* and *pTRV2* (Liu *et al.*, 2002) vectors were used for VIGS experiments. The gene-specific fragment for *GhMYB108* (Supplementary Fig. S1) or *GhCML11* was inserted into *pTRV2*. *Agrobacterium* cultures (OD_600_=1.5) harboring *pTRV1* and *pTRV2-GhMYB108* or *pTRV2-GhCML11* were mixed at a 1:1 ratio and agroinoculated into cotton plants by vacuum infiltration as described by Qu *et al.* (2012). Alternatively, cotyledons of seedlings were transfected with the mixture using a needle-less syringe as described by Gao *et al.* (2011). *GhCLA1* was used as the positive control (Gao *et al.*, 2011).

### Yeast two-hybrid (Y2H) assay

Y2H assay was performed according to the instructions of the manufacturer of the Matchmaker Gold Yeast Two-Hybrid System (Clontech). The ORF of *GhMYB108* was cloned into the BD vector *pGBKT7* to construct *BD-GhMYB108* as bait. The ORF of *GhCML11* was inserted into the AD vector *pGADT7* to produce the prey constructs. Yeast strain AH109 cells co-transformed with the bait and prey constructs were plated onto SD/–Leu/–Trp DO (DDO) medium. After growth at 30 °C for 72h, the independent colony with the same size was transferred to SD/–Leu/–Trp/–Ade/–His DO (QDO) medium supplemented with 5mM 3-aminotriazole (3-AT). The interactions were also visually detected using an X-gal filter assay.

### Pull-down assay

MBP–GhMYB108 and GST–GhCML11 fusion proteins were expressed and purified as described above. The *in vitro* protein–protein interaction assay was carried out according to the ProFound Pull-Down GST Protein Kit. The eluted proteins were separated on an SDS–PAGE gel and detected by western blot using anti-GST or anti-MBP antibodies (1:4000; Sungene Biotechnology).

### Firefly luciferase complementation imaging (LCI) assay

The ORF of *GhMYB108* was inserted into the *pCAMBIA-NLuc* vector. The ORF of *GhCML11* was cloned into the *pCAMBIA-CLuc* vector. Equal amounts of *Agrobacterium* cultures containing CLuc and NLuc constructs were mixed, and then co-infiltrated into *N. benthamiana* leaves. The infiltrated leaves were analyzed for relative Luc activity 48h after infiltration using a low-light cooled charge-coupled device camera (Night owl LB985, Germany). Quantitative analysis was performed using the IndiGo software (Berthold Technologies).

### Transient expression assay

The transient expression assay was performed as described by Shang *et al.* (2010). The *GhPR4*, *GhPR5*, and *GhPDF1.2* promoters were fused with the *Luc* reporter gene in the plant binary vector *pGWB435* (Invitrogen) to generate the reporter constructs *GhPR4*_*pro*_ *:Luc*, *GhPR5*_*pro*_ *:Luc*, and *GhPDF1.2*_*pro*_ *:Luc*, respectively. The ORF of *GhMYB108* was cloned into the *pBI121* vector to generate the effector construct *35S*_*pro*_ *:GhMYB108*, and the ORF of *GhCML11* was cloned into the *pPZP111* vector to generate the effector construct *35S*_*pro*_ *:GhCML11*. The *Agrobacterium* strains containing different constructs were infiltrated into *N. benthamiana* leaves. The infiltrated plants were incubated for 48h at 23 °C. Image capture used the low-light cooled charge-coupled device camera, and calculation of the relative Luc activity was performed using the IndiGo software.

### Ca^2+^-dependent mobility shift assay

The Ca^2+^-dependent mobility shift assay was conducted according to Garrigos *et al.* (1991). The GST-tag in GST–GhCML11 was cleaved using PreScission Protease (GE Healthcare), and GhCML11 proteins were electrophoresed on a 15% SDS–PAGE gel in the presence of either 5mM CaCl_2_ or 5mM EGTA, and then visualized by Coomassie brilliant blue staining.

### [Ca^2+^]_cyt_ staining

Staining of cytosolic Ca^2+^ was performed as described by Zhang *et al.* (1998). Cotton seedlings were grown under hydroponic conditions. *Agrobacterium* cultures harboring *pTRV1* and *pTRV2* (control), *pTRV2-GhMYB108*, or *pTRV2-GhCML11* were mixed at a 1:1 ratio and agroinoculated into cotton plants by vacuum infiltration, and then the plants were transferred to steam-sterilized vermiculite. After 2 weeks, seedlings were gently uprooted and rinsed with sterile water, and then placed in sterile water for 24h to adapt to hydroponic conditions. The roots were infected by spore suspensions (10^6^ spores ml^−1^). The cotton roots were then loaded with Ca^2+^-sensitive fluorescent dye Fluo-4/AM (Invitrogen) at 4 °C for 2h followed by 2h at 25 °C in the dark. The fluorescence of the cotton root cells was visualized with a confocal microscopy. The fluorescence intensity of root cells was determined using Leica LAS AF Lite software.

### Transcriptome analysis

For transcriptome analysis, total RNAs were extracted from control (*TRV:00*) and *GhMYB108*-silenced (*TRV:GhMYB108*) plants. The library construction and Illumina sequencing were conducted by BGI (http://www.genomics.cn/en/index). After eliminating the adaptors and low-quality sequences, the sequence reads were used for further analysis. Genes with differentially expressed transcripts [fold change ≥2 and false discovery rate (FDR) <0.001] in *GhMYB108*-silenced plants compared with control plants were identified. The accession number of the raw transcriptomic data is SRP067059.

### Accession numbers

Sequence data for the genes described in this study can be found in the GenBank/EMBL database under the following accession numbers: *GhMYB108* (KT281917), *GhCML11* (KT281918), *AtPDF1.2* (AT5G44420), *AtPR4* (AT3G04720), *AtPR5* (AT1G75040), *AtWRKY18* (AT4G31800), *AtWRKY33* (AT2G38470), *AtWRKY50* (AT5G26170), *AtbHLH87* (AT3G21330), *AtWAK2* (AT1G21270), *AtFLS2* (AT5G46330), *AtBAK1* (AT4G33430), *AtLYK4* (AT2G23770), *AtANP3* (AT3G06030), *AtMKK4* (AT1G51660), *AtMKK6* (AT5G 56580), *AtAHK4* (AT2G01830), *AtRLP12* (AT1G71400), *AtCYP82G1* (AT3G25180), *AtCYP707A1* (AT4G19230), *AtRGA2* (AT1G14920), *AtRPP13* (AT3G46530), *AtH2A* (AT5G54640), *AtSOT17* (AT1G18590), and *AtPUB23* (AT2G35930).

## Results

### Expression of *GhMYB108* responds to *V*. *dahliae* infection

In our ongoing studies of the defense-related genes acting in the response against cotton Verticillium wilt, we frequently noticed the presence of MBS (MYB-binding site) *cis*-elements in the promoters of the defense-responsive genes. To investigate the role of cotton *MYB* genes in defense against *V*. *dahliae* infection, we first conducted a database search and randomly selected six candidate *MYB* genes from different subfamilies to compare the pathogen-responsive expression of the *MYB* genes in upland cotton. Among these *MYB* genes, one gene (*GhMYB108*) showed strong induction of transcription upon pathogen inoculation (Supplementary Fig. S2). Since two members of this subfamily of *MYB* genes were shown to participate in defense against fungus infection in Arabidopsis or wheat (Mengiste *et al.*, 2003; Z. Zhang *et al.*, 2012), we focused our study on the functional mechanism of the *GhMYB108* gene in protection against *V. dahliae* infection in cotton.

**Fig. 1.**
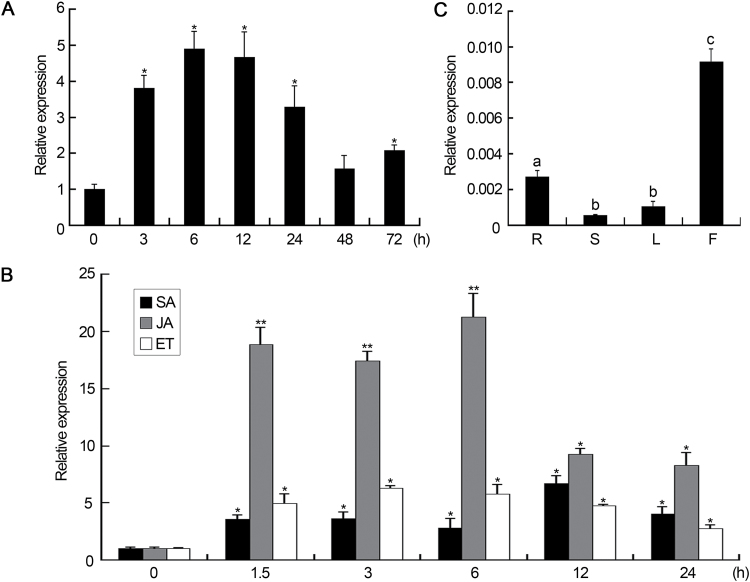
Expression pattern of the *GhMYB108* gene in cotton plants. (A) Accumulation of *GhMYB108* transcripts in cotton roots in response to *V. dahliae* infection. Error bars represent the SD of three biological replicates. Asterisks indicate statistically significant differences, as determined by Student’s *t*-test (*P<0.05). (B) Expression of *GhMYB108* after treatments with salicylic acid, jasmonic acid, and ethylene. Asterisks indicate statistically significant differences, as determined by Student’s *t*-test (*P<0.05, **P<0.01). (C) qRT-PCR analysis of *GhMYB108* expression in root (R), stem (S), leaf (L), and flower (F) of cotton plants. Different letters indicate statistically significant differences at *P*<0.05 (Student’s *t*-test, three biological replicates).

qRT-PCR analysis was performed to measure the time course of pathogen-responsive expression of *GhMYB108.* As shown in Fig. 1A, the expression of *GhMYB108* increased in roots after *V. dahliae* infection and reached a maximal level at 6h post-inoculation. Next, *GhMYB108* expression was analyzed after treatment with the defense-related signaling molecules salicylic acid, jasmonic acid, and ethylene. The results showed that these three signaling molecules enhanced the accumulation of *GhMYB108* transcripts to different extents (Fig. 1B), supporting the idea that *GhMYB108* could be involved in defense against *V*. *dahliae* invasion in cotton plants. Expression of *GhMYB108* was also examined in various organs of the cotton plant. *GhMYB108* transcripts accumulated to a higher level in the root, which is the site of the *V. dahliae* invasion, as compared with the stem and leaf (Fig. 1C). The expression of *GhMYB108* was the highest in flowers, implying that *GhMYB108* may also function in flower development.

### GhMYB108 is a functional transcription activation factor

**Fig. 2.**
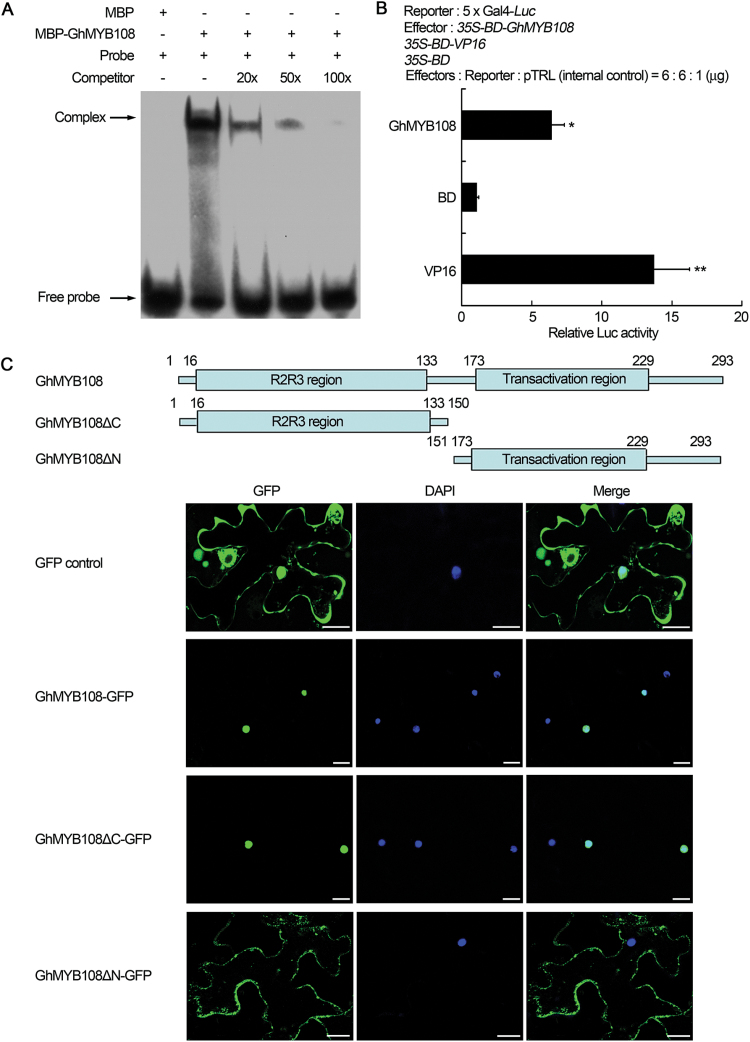
Transcriptional activity of GhMYB108 and subcellular localization of GhMYB108–GFP fusion proteins. (A) EMSA analysis of the binding of GhMYB108 to the MBS *cis*-elements. GhMYB108 proteins were incubated with biotin-labeled probe (2× TAACGGAC) in the absence or presence of a 20-, 50-, or 100-fold excess of unlabeled competitor. (B) Transcriptional activation activity of GhMYB108 in Arabidopsis protoplasts. The empty vector *pRT-BD* and *pRT-BD-VP16* were used as negative and positive control, respectively. Error bars represent the SD of three biological replicates. Asterisks indicate statistically significant differences, as determined by Student’s *t*-test (*P<0.05, **P<0.01). (C) Subcellular localization of intact and truncated fusion proteins. GFP, GhMYB108–GFP, GhMYB108ΔC–GFP, and GhMYB108ΔN–GFP fusion proteins were transiently expressed in *N. benthamiana* leaves. GFP fluorescence was visualized by confocal microscopy. Numbers represent amino acid residues. Scale bars=20 μm. (This figure is available in colour at *JXB* online.)

EMSA was used to test the DNA-binding activity of GhMYB108. The results showed that GhMYB108 proteins and labeled probe could form a complex, and addition of non-labeled probes dramatically reduced the observed DNA binding activity, indicating that GhMYB108 could bind specifically to the MBS *cis*-element (Fig. 2A).

The TF activity of GhMYB108 was examined using the DLR assay in Arabidopsis protoplasts. Isolation and transformation of Arabidopsis protoplasts were carried out as described by He *et al.* (2007). Compared with the negative control, the protoplasts harboring GhMYB108 showed significantly higher luciferase activity (Fig. 2B), indicating that GhMYB108 can activate the transcription of the *Luc* reporter gene *in vivo*.

### The region containing the R2R3 domain is required for the nuclear localization of GhMYB108

To examine the nuclear distribution of GhMYB108, *Agrobacterium* cells transformed with the *GhMYB108-GFP* fusion and *GFP* control constructs were infiltrated into *N. benthamiana* leaves. Transiently expressed GhMYB108–GFP proteins were mainly localized in the nucleus, whereas GFP control was diffusely localized throughout the cytoplasm and nucleus (Fig. 2C).

As no nuclear localization signal was found in the GhMYB108 protein sequence, we wished to know which region of the protein might be responsible for its nuclear distribution. To this end, plasmids harboring cDNA fragments encoding either C-terminus-deleted *GhMYB108-GFP* (*GhMYB108ΔC-GFP*) or N-terminus-deleted *GhMYB108-GFP* (*GhMYB108ΔN-GFP*) were constructed, and *Agrobacterium* cells transformed with these constructs were separately infiltrated into *N. benthamiana* leaves. GhMYB108ΔC–GFP proteins were localized in the nucleus, while GhMYB108ΔN–GFP proteins were distributed in the cytoplasm without entry into the nucleus (Fig. 2C). These results indicate that the region containing the R2R3 domain of GhMYB108 is required for the nuclear localization of GhMYB108.

### Silencing of *GhMYB108* in cotton plants impairs the tolerance to *V. dahliae*

**Fig. 3.**
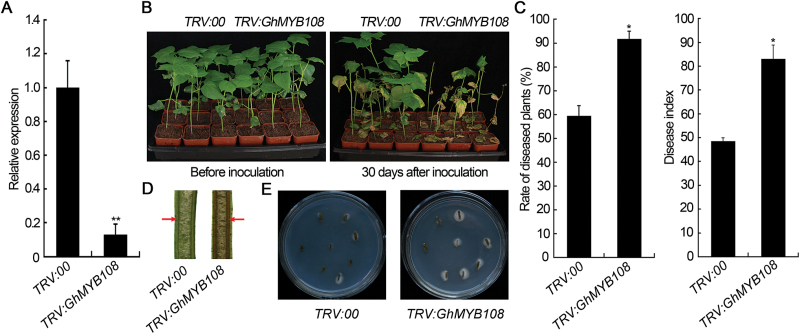
Increased susceptibility of *GhMYB108*-silenced cotton plants to *V. dahliae*. (A) Analysis of *GhMYB108* expression levels. Total RNAs were extracted from leaves of cotton plants at 14 d post-agroinfiltration, and the expression level of *GhMYB108* in VIGS plants was compared with that of the control plant (*TRV:00*). Asterisks indicate statistically significant differences, as determined by Student’s *t*-test (**P<0.01). (B) Disease symptoms of control (*TRV:00*) and *GhMYB108*-silenced (*TRV:GhMYB108*) plants infected by *V. dahliae*. (C) Rate of diseased plants and disease index of the control and *GhMYB108*-silenced plants. Error bars represent the SD of three biological replicates (*n*≥30). Asterisks indicate statistically significant differences, as determined by Student’s *t*-test (*P<0.05). (D) Comparison of a longitudinal section of stem between control and *GhMYB108*-silenced cotton plants 20 d after *V. dahliae* infection. Arrows indicate the vascular part of the stem. (E) Fungal recovery assay. The stem sections from cotton plants 20 d after *V*. *dahliae* infection were plated on potato dextrose agar medium. Photos were taken at 6 d after plating. The number of stem sections on which the fungus grew showed the extent of fungal colonization. (This figure is available in colour at *JXB* online.)

The VIGS strategy was employed to study the role of *GhMYB108* in response to *V. dahliae*. *GhCLA1* (produces an albino phenotype when silenced) was used as the positive control. At 14 d after agroinfiltration with the *GhCLA1* construct, the cotton leaves displayed the expected photobleaching phenotype (Supplementary Fig. S3), showing that the VIGS system worked well under our experimental conditions. Compared with the control, expression of *GhMYB108* was obviously reduced in *GhMYB108*-silenced plants (Fig. 3A). To see if off-target silencing of other *MYB* genes was caused in the VIGS plants, we examined the transcription levels of six potential off-target genes, and the results showed that expression of these genes was not affected (Supplementary Figs S1, S4).

*GhMYB108*-silenced and control plants were challenged with *V. dahliae*. Before inoculation of the pathogen, no significant phenotype change was observed in VIGS plants as compared with the control plants (Fig. 3B, left panel). At 20 d after infection, *GhMYB108*-silenced plants began to show increased susceptibility to *V. dahliae*, with more severe wilting and yellowing symptoms than control plants. After a further 10 d, the disease symptoms of *GhMYB108*-silenced plants became more evident (Fig. 3B, right panel). The rate of diseased plants and the disease index were higher for the *GhMYB108*-silenced plants than for the control plants (Fig. 3C). In addition, the vascular tissue of *GhMYB108*-silenced cotton plants turned brown 20 d after infection with *V. dahliae,* but that of the control plants was not chlorotic when tested at the same time (Fig. 3D). These observations indicated that silencing of *GhMYB108* enhanced the susceptibility to *V. dahliae*. The fungal recovery assay from stem sections of inoculated cotton plants also confirmed the role of *GhMYB108* in cotton defense against *V. dahliae* (Fig. 3E).

### Overexpression of *GhMYB108* enhances tolerance to *V. dahliae* in transgenic Arabidopsis plants

**Fig. 4.**
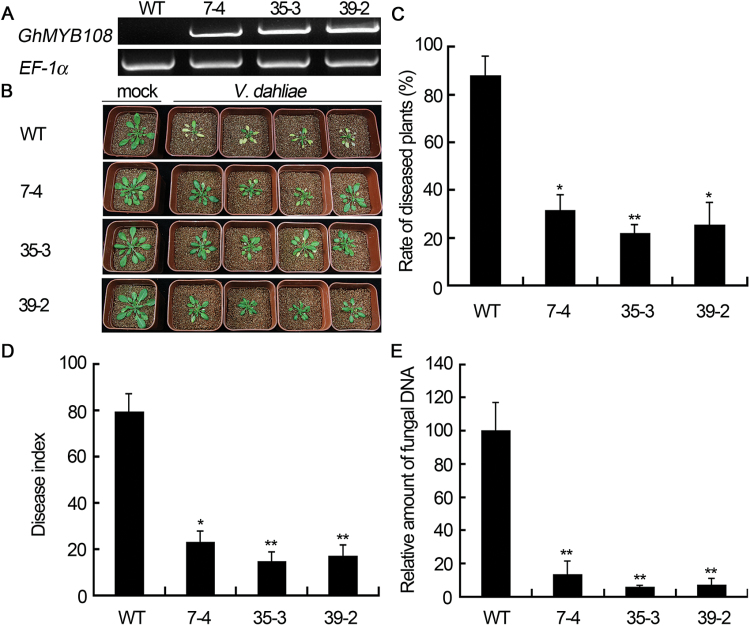
Enhanced disease tolerance of Arabidopsis plants overexpressing *GhMYB108*. (A) Expression levels of *GhMYB108* in WT (wild-type) and transgenic Arabidopsis lines (7-4, 35-3, and 39-2). (B) Symptoms of WT and *GhMYB108* transgenic plants inoculated with *V. dahliae* for 22 d. (C and D) Rate of diseased plants and disease index of WT and transgenic plants. Error bars indicate the SD of three biological replicates with 36 plants per repeat. (E) Quantification of fungal biomass. Real-time PCR analysis was conducted to compare the transcript levels between the *ITS* gene (as a measure for fungal biomass) of *V. dahliae* and the *Rubisco* gene of Arabidopsis (for equilibration) at 22 d post-inoculation. Relative amounts of fungal DNA were set to 100% for the WT. Asterisks indicate statistically significant differences, as determined by Student’s *t*-test (*P<0.05, **P<0.01). (This figure is available in colour at *JXB* online.)

A gain-of-function approach was also employed to study the function of GhMYB108 in the defense response. Due to technical difficulties and long duration of cotton transformation, we used the model plant Arabidopsis, which is also a host for *V. dahliae*. Three lines (7-4, 35-3, and 39-2) of transgenic plants with different expression levels of *GhMYB108* were selected for further study (Fig. 4A). Arabidopsis plants at ~4 weeks old were inoculated with *V. dahliae*. Fifteen days after inoculation, the leaves of Arabidopsis began to show wilting and yellowing symptoms, and the plants grew stunted and short. Compared with the wild type, the transgenic plants showed much weaker symptoms at 22 d post-inoculation (Fig. 4B). The rate of diseased plants and disease index of the transgenic plants were significantly lower than those of the wild-type plants (Fig. 4C, D), showing that ectopic overexpression of *GhMYB108* conferred increased disease tolerance to *V. dahliae* in Arabidopsis plants. To verify the observed phenotype further, the fungal biomass was measured by real-time PCR. Less fungal DNA was measured in transgenic plants than in wild-type plants (Fig. 4E), supporting the conclusion that *GhMYB108*-transgenic plants were more tolerant to *V. dahliae* infection.

In addition to *V. dahliae*, we also inoculated the *GhMYB108*-overexpressing Arabidopsis plants with two other pathogens, the bacterium *Pst* DC3000 and the fungus *B. cinerea*. The results showed that these plants were less susceptible to *B. cinerea* as compared with the wild type, but similar disease symptoms were found between the wild-type and transgenic plants infected with *Pst* DC3000, indicating that *GhMYB108* overexpression rendered the transgenic Arabidopsis plants specifically more tolerant to the fungal pathogen (Supplementary Fig. S5).

### GhMYB108 interacts with GhCML11

**Fig. 5.**
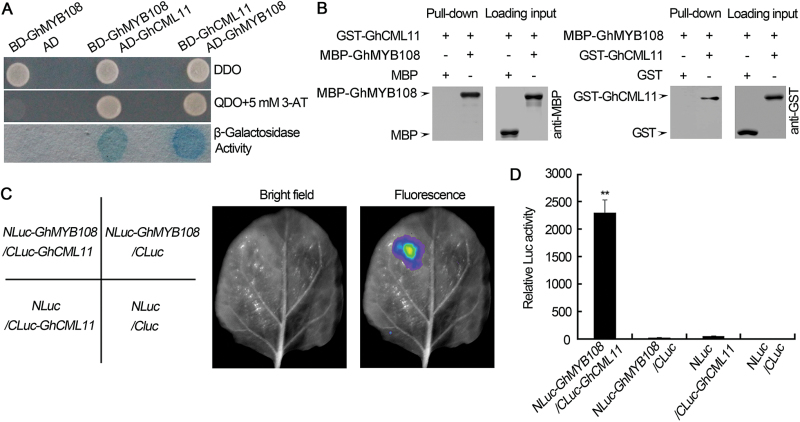
Interaction of GhMYB108 and GhCML11 proteins. (A) Yeast two-hybrid assay to detect interaction between GhMYB108 and GhCML11. The yeast strain containing the indicated plasmids was grown on SD/–Leu/–Trp DO (DDO) plates and SD/–Leu/–Trp/–Ade/–His DO (QDO) plates (containing 5mM 3-AT) for 3 d. Interaction of GhMYB108 with the AD domain in the pGADT7 empty vector was used as a negative control. (B) Pull-down assay. GST–GhCML11 fusion protein was used as bait, and MBP–GhMYB108 fusion protein was used as prey. Alternatively, MBP–GhMYB108 fusion protein was used as bait, and GST–GhCML11 fusion protein was used as prey. The anti-MBP and anti-GST antibodies were used to detect bait and prey proteins. MBP and GST proteins were used as negative controls. (C) LCI analysis of the interaction between GhMYB108 and GhCML11. *Agrobacterium* strains containing the indicated pairs were co-expressed in *N. benthamiana*. The luminescent signal was collected at 48h after infiltration. (D) Quantification of relevant Luc activities in (C). Error bars represent the SD of three biological replicates. Asterisks indicate statistically significant differences, as determined by Student’s *t*-test (**P<0.01). (This figure is available in colour at *JXB* online.)

The Y2H system was employed to identify protein(s) that may interact with GhMYB108. Screening the cDNA library of cotton roots infected by *V. dahliae* identified a cDNA that encodes a CaM-like protein (designated GhCML11). Direct Y2H assays confirmed the interaction between the two proteins (Fig. 5A). A pull-down assay was performed to verify further the interaction of the two proteins (Fig. 5B). Equal amounts of lysates containing GST–GhCML11 were incubated with immobilized MBP or MBP–GhMYB108 proteins. As expected, GhCML11 bound to GhMYB108, but not to the control MBP proteins. Subsequently, lysates containing MBP–GhMYB108 were incubated with immobilized GST or GST–GhCML11 proteins. GhMYB108 bound to GhCML11, but not to the control GST proteins. These results confirmed that GhMYB108 and GhCML11 could interact.

To verify the interaction of the two proteins *in planta*, an LCI assay (Chen *et al.*, 2008) was conducted. As shown in Fig. 5C and D, strong Luc activity was detected in *N. benthamiana* leaves, but no significant Luc activity was detected in the negative controls.

**Fig. 6.**
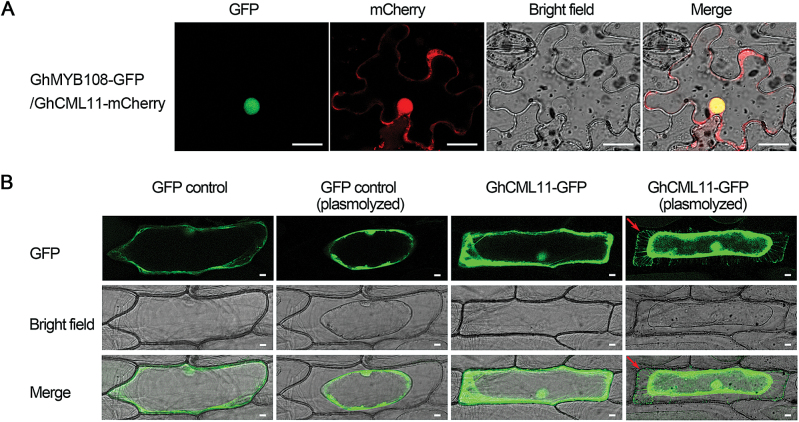
Subcellular localization of GhCML11 proteins. (A) Co-localization of GhMYB108 and GhCML11 in the nucleus. *Agrobacterium* strains containing the indicated pair of GhMYB108-GFP and GhCML11-mCherry were co-expressed in *N. benthamiana*. The signal was visualized with confocal microscopy. Scale bars=20 μm. (B) Localization of GhCML11 transiently expressed in onion epidermal cells. The two left-hand panels show the cells containing the empty vector before and after plasmolysis. The two right-hand panels show the cells harboring the GhCML11–GFP construct before and after plasmolysis. Arrows indicate the cell wall region after plasmolysis. Scale bars=20 μm. (This figure is available in colour at *JXB* online.)

Since GhCML11 interacts with GhMYB108, we investigated whether the subcellular localization of GhCML11 was similar with GhMYB108. *Agrobacterium* cells containing *GhMYB108-GFP* and *GhCML11-mCherry* were co-infiltrated into *N. benthamiana* leaves. Indeed, GhCML11 co-localized with GhMYB108 in the nucleus (Fig. 6A). In addition to the nucleus, we also noticed GhCML11 in the periphery of the *N. benthamiana* pavement cells (Fig. 6A). To see this subcellular localization of GhCML11 more clearly, we bombarded the *GhCML11-GFP* construct into onion epidermal cells and used plasmolysis to examine the plasma membrane and apoplast. GhCML11–GFP fluorescence was observed in both the nucleus and cytoplasm (Fig. 6B). Interestingly, we found that some GhCML11 proteins remained in the apoplast after plasmolysis. However, no free GFP signal was detected in the extracellular region after plasmolysis in the cells transformed with GFP alone. Thus, as reported for some CaMs in other plants (Cui *et al.*, 2005; Wang *et al.*, 2013), GhCML11 is probably also an apoplastic protein. As a protein that lacks a signal peptide but can be secreted from the cell independent of the endoplasmic reticulum/Golgi system can be defined as a non-classically secreted protein (Nickel and Rabouille, 2009; Drakakaki and Dandekar, 2013), GhCML11 belongs to such a protein group based on its sequence and localization. Indeed, GhCML11 is predicted to be a non-classically secreted protein by the online software http://www.cbs.dtu.dk/services/SecretomeP-1.0/.

### GhCML11 promotes the transcriptional function of GhMYB108

**Fig. 7.**
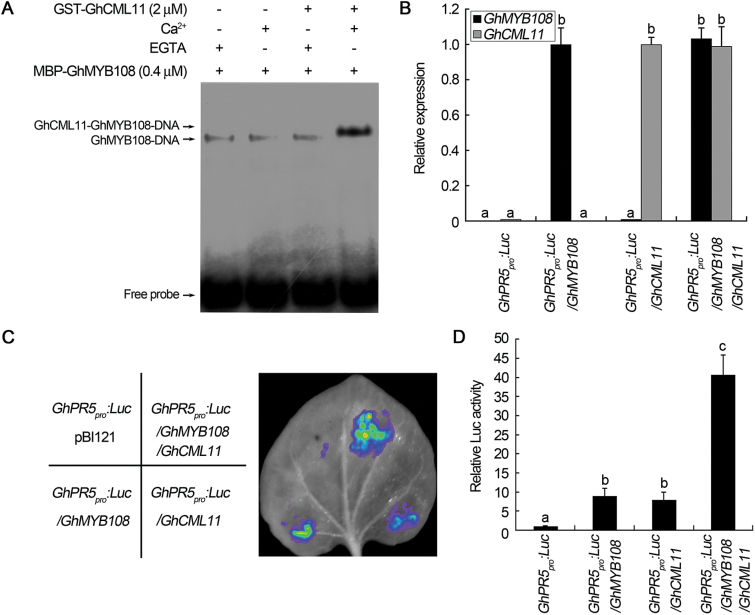
GhCML11 promotes transcriptional activity of GhMYB108. (A) Effect of GhCML11 on the DNA binding activity of GhMYB108. (B) qRT-PCR analysis of *GhMYB108* and *GhCML11* expression in the infiltrated *N. benthamiana* leaves transformed with the indicated constructs in (C). Different letters indicate statistically significant differences at *P*<0.01 (Student’s *t*-test, *n*≥15, three biological repeats). (C) Effect of GhCML11 on the transcription factor activity of GhMYB108. Luminescence imaging was performed 48h after co-infiltration. (D) Quantitative analysis of luminescence intensity in (C). Different letters indicate statistically significant differences at *P*<0.05 (Student’s *t*-test, *n*=30, three biological repeats).

Since GhMYB108 acts as a TF, the interaction between GhCML11 and GhMYB108 may have an effect on its activity. To test this possibility, EMSA was performed in the presence of GhCML11. As shown in Fig. 7A, GhMYB108 bound to the MBS *cis*-elements and formed a band representing the DNA–protein complex; when GhCML11 and Ca^2+^ were present in the reaction simultaneously, a supershifted band with markedly enhanced intensity appeared. When GhCML11 was included in the reaction without addition of Ca^2+^, no effect was observed on the DNA binding activity of GhMYB108 either. The result indicated that the DNA binding activity of GhMYB108 was enhanced by its interaction with GhCML11 in a Ca^2+^-dependent manner *in vitro*. The EMSA was conducted to determine the Ca^2+^ binding property of GhCML11. It is known that CaMs undergo conformational changes and exhibit an increase in their electrophoretic migration rates after binding Ca^2+^ (Garrigos *et al.*, 1991; Wang *et al.*, 2015). As shown in Supplementary Fig. S6, the mobility of GhCML11 was increased in the presence of Ca^2+^, demonstrating that GhCML11 is a functional Ca^2+^-binding protein.

We next conducted an *in vivo* test to see if the effect of GhCML11 on GhMYB108 DNA binding activity reflects its role in the TF activity of GhMYB108. As it was reported that a plant MYB could bind to the promoter sequence of *PR5* (thaumatin-like protein) and regulate its transcription (Kenton *et al.*, 2000; Z. Zhang *et al.*, 2012), we performed a transient expression assay by using the promoter sequence of a cotton *PR5* gene to drive the expression of the reporter gene with or without the presence of GhCML11 (Fig. 7B–D). First, the binding of GhMYB108 to the *GhPR5* promoter was tested by EMSA. As shown in Supplementary Fig. S7C, GhMYB108 bound to the *GhPR5* promoter efficiently. The *GhPR5* promoter was then fused to the *Luc* reporter gene (*GhPR5*_*pro*_ *:Luc*) and infiltrated into *N. benthamiana* leaves. Two days later, the expression of *GhMYB108* and *GhCML11* was confirmed by qRT-PCR (Fig. 7B) and *Luc* expression was examined. The results showed that the *GhPR5* promoter drove *Luc* expression weakly on its own, but co-expression of *GhPR5*_*Pro*_ *:Luc* with GhMYB108 created an obvious increase in Luc activity, indicating that GhMYB108 activated the expression of *Luc* driven by the *PR5* promoter. Luc activity was also enhanced when *35S:GhCML11* was co-transformed with *GhPR5*_*Pro*_ *:Luc*, probably caused by endogenous GhMYB108 homolog(s) in *N. benthamiana*, which might act co-operatively with GhCML11 and promote the *GhPR5* promoter activity. Co-expression of the *GhPR5*_*Pro*_ *:Luc* reporter with GhMYB108 and GhCML11 led to much stronger Luc intensity than in the cells injected with the *GhPR5*_*Pro*_ *:Luc* reporter and GhMYB108 (Fig. 7C, D), demonstrating that GhCML11 could promote the transcriptional activation activity of GhMYB108 in plant cells.

### GhMYB108 regulates the transcription of *GhCML11*

**Fig. 8.**
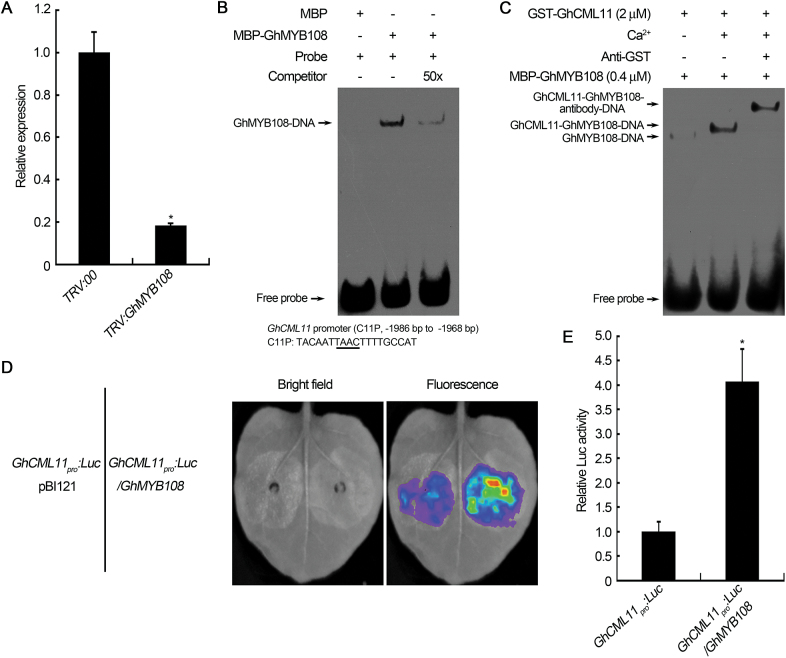
GhMYB108 regulates the transcription of *GhCML11*. (A) Expression analysis of *GhCML11* in control (*TRV:00*) and *GhMYB108*-silenced (*TRV:GhMYB108*) plants. Asterisks indicate statistically significant differences, as determined by Student’s *t*-test (*P<0.05). (B) EMSA of the binding of GhMYB108 to the promoter of *GhCML11*. The underlined sequence indicates the core motif of the MYB-binding site. (C) Analysis of the effect of GhCML11 proteins on the binding activity of GhMYB108 to the *GhCML11* promoter. Anti-GST antibody against GST-tagged GhCML11 was added in the reaction to detect the presence of GhCML11 in the GhMYB108–DNA complexes. (D) Activation of *GhCML11* transcription by GhMYB108. Luminescence imaging was performed 48h after co-infiltration of *N. benthamiana* leaves with equal amounts of *Agrobacterium* cells containing the indicated constructs on the left panel. (E) Quantitative analysis of luminescence intensity in (D). Error bars represent the SD (*n*=30) of three biological replicates. Asterisks indicate statistically significant differences, as determined by Student’s *t*-test (*P<0.05). (This figure is available in colour at *JXB* online.)

In *GhMYB108*-silenced cotton plants, the expression of *GhCML11* was also suppressed (Fig. 8A). This raised the possibility that GhMYB108 may affect the transcription of *GhCML11*. To test this, the promoter sequence of *GhCML11* was isolated and subjected to EMSA analysis. As shown in Fig. 8B, GhMYB108 protein could form a complex with the promoter sequence of *GhCML11*, and non-labeled probes considerably decreased the binding of GhMYB108 proteins to the labeled probes, indicating that GhMYB108 could specifically bind to the tested sequence of the *GhCML11* promoter. As it was observed that binding of GhMYB108 to MBS *cis*-elements could be increased by GhCML11, we also tested if GhCML11 could enhance the binding of GhMYB108 to its own promoter. As shown in Fig. 8C, a supershifted signal with enhanced intensity appeared when GhCML11 proteins and Ca^2+^ were added in the reaction; when an anti-GST antibody was added to the binding mixture, the band was shifted even further, indicating that GhCML11 was present in the supershifted band.

To investigate further whether *GhMYB108* could activate the transcription of *GhCML11* in plant cells, the promoter of *GhCML11* was inserted into the vector pGWB435 with the *Luc* reporter gene. Co-expression of the *GhCML11* promoter fused to the *Luc* reporter gene with *35S:GhMYB108* showed an obvious increase in Luc activity as compared with the control (Fig. 8D, E), indicating that GhMYB108 activated the expression of *Luc* driven by the *GhCML11* promoter.

### Calcium signaling is active in response to *V. dahliae* infection in cotton

**Fig. 9.**
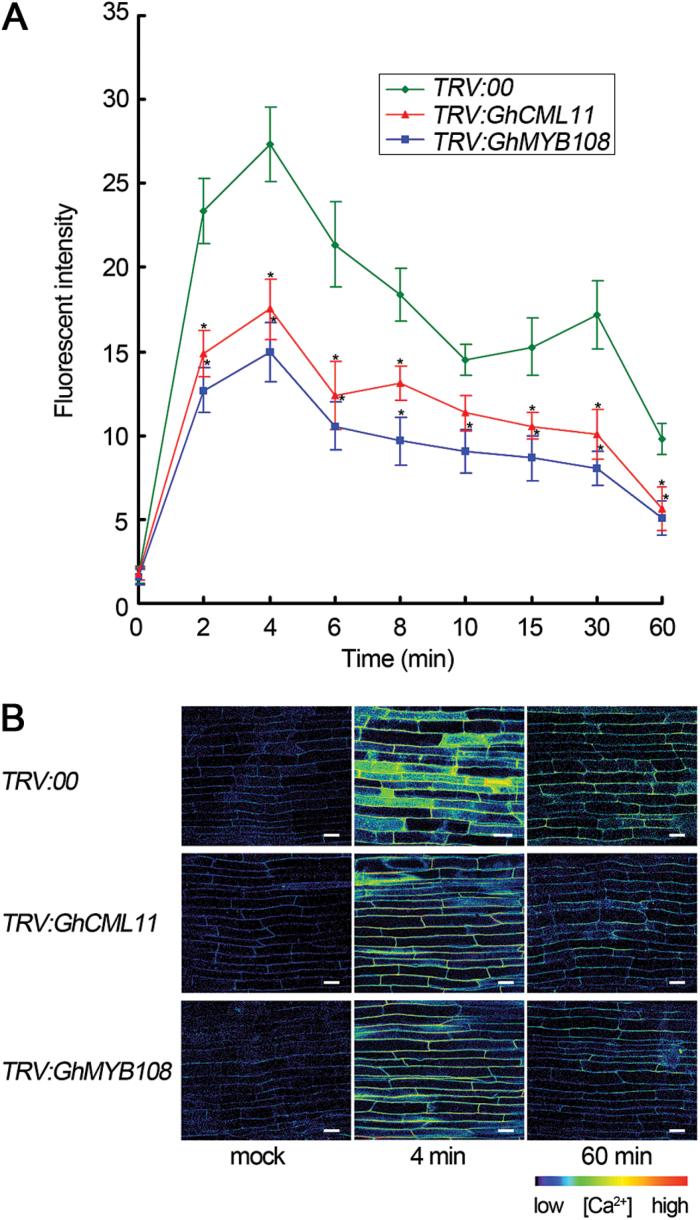
Ca^2+^ levels in cytosol of root cells in control, *GhMYB108*-silenced, and *GhCML11*-silenced cotton plants. (A) Change in fluorescent intensity of control, *GhMYB108*-, and *GhCML11*-silenced cotton root cells treated with Fluo-4/AM at the indicated time points after inoculation with *V. dahliae*. Error bars represent the SD (*n*≥10) of three biological replicates. Asterisks indicate statistically significant differences, as determined by Student’s *t*-test (*P<0.05). (B) Fluorescence images of cotton root cells at 0, 4, and 60min post-inoculation with *V. dahliae*. The fluorescence signals were visualized by confocal microscopy. Scale bars=20 μm.

Ca^2+^ plays an important role in plant immune responses (Lecourieux *et al.*, 2006). To gain more insight into the involvement of Ca^2+^-mediated signaling in the cotton defense response against *V*. *dahliae*, we performed a time course experiment to assess the change of [Ca^2+^]_cyt_ in response to *V*. *dahliae* infection. The cotton root cells were loaded with the Ca^2+^ indicator Fluo-4/AM. The fluorescence intensity in the root cells of control plants increased significantly after inoculation with the pathogen, reaching a peak at 4min and then decreasing quickly (Fig. 9). The result indicated that Ca^2+^ influx into the cytosol occurred in response to *V*. *dahliae* infection.

The fluorescence intensity in the root cells of *GhMYB108*-silenced and *GhCML11*-silenced plants was compared with that of the control plants. Before *V*. *dahliae* infection, the fluorescence intensity in *GhMYB108*- and *GhCML11*-silenced root cells was similar to that of control root cells, but it increased relatively less upon pathogen inoculation, indicating that the influx of [Ca^2+^]_cyt_ upon *V*. *dahliae* infection was influenced in these cells (Fig. 9). These results show that Ca^2+^ influx into the cytosol occurs in response to *V*. *dahliae* invasion and the expression levels of *GhCML11* and *GhMYB108* had an impact on this process.

### Transcriptomic analysis of genes affected in *GhMYB108*-silenced cotton plants

**Fig. 10.**
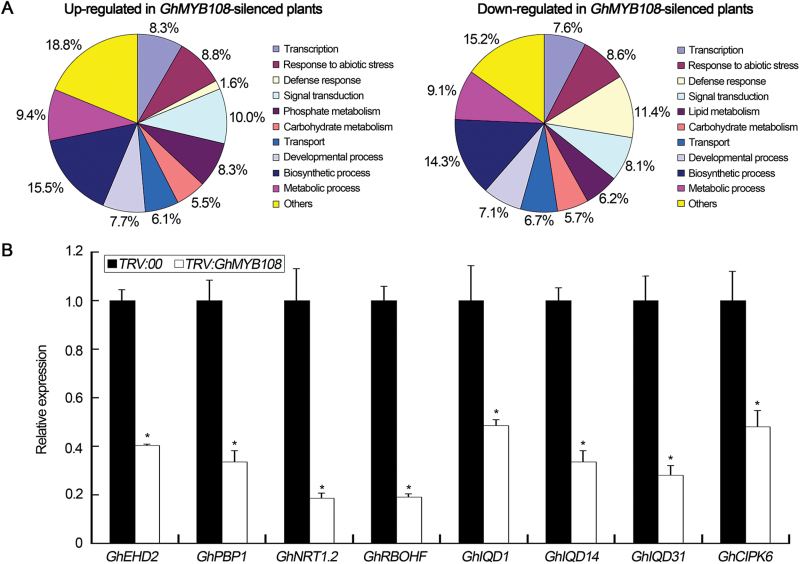
Transcript profiling analysis of differentially expressed genes in the *GhMYB108*-silenced cotton plants. (A) Functional classification of genes up- or down-regulated in *GhMYB108*-silenced cotton plants. The percentage of each category of up-regulated or down-regulated genes indicates the number of genes in that category relative to the 181 annotated up-regulated or 210 annotated down-regulated genes. (B) The expression levels of calcium signaling genes between control (*TRV:00*) and *GhMYB108*-silenced (*TRV:GhMYB108*) plants. These genes included Ca^2+^-binding protein genes *GhEHD2* (EPS15 homology domain protein), *GhPBP1* (PINOID-binding protein), *GhNRT1.2* (Nitrate transporter1.2), *GhRBOHF* (Respiratory burst oxidase homolog protein), calmodulin-binding protein genes *GhIQD1*, *GhIQD14*, and *GhIQD31* (IQ-domain protein), and the CBL-binding protein gene *GhCIPK6.* Error bars represent the SD of three biological replicates. Asterisks indicate statistically significant differences, as determined by Student’s *t*-test (*P<0.05).

Comparative transcriptome analysis was employed to identify genes possibly regulated by *GhMYB108*. A total of 391 differentially expressed genes (fold change ≥2 and FDR <0.001) were identified, of which 181 genes were up-regulated and 210 genes were down-regulated (Supplementary Table S2). Among the differentially expressed genes, a large number were involved in the biological processes of transcriptional regulation, signal transduction, developmental process, biosynthesis, and metabolism (Fig. 10A). In accordance with the above results on the relationship between GhMYB108 and Ca^2+^/GhCML11, several calcium signaling genes were down-regulated in *GhMYB108*-silenced cotton plants (Fig. 10B).

Among the identified differentially expressed genes, 23 defense-related genes were inhibited in *GhMYB108*-silenced plants (Supplementary Table S3). The expression of these genes in *GhMYB108*-silenced cotton plants was then evaluated by qRT-PCR, which verified the down-regulation of these genes (Supplementary Fig. S8). We also analyzed the expression of these genes in *GhMYB108*-overexpressing Arabidopsis (Supplementary Figs S7B, S9), and observed that 14 out of 23 genes were up-regulated. These results indicate that there is a correlation between expression of *GhMYB108* and these defense-related genes. As a previous study suggested that MYB may bind to the promoter sequences of some defense-related genes, we analyzed the promoter sequences of these 14 genes for the presence of MBS *cis*-elements, and found that several of these genes harbored the MBS motifs in their promoters. Of these, we chose *PDF1.2* (defensin-like gene), *PR4*, and *PR5* (see above), which showed altered expression in both *GhMYB108*-overexpressing and *GhMYB108*-silenced plants (Supplementary Fig. S7A, B), and tested the binding of GhMYB108 to their promoter sequences by EMSA (Supplementary Fig. S7C, D). GhMYB108 could bind to the promoter fragments of these three genes. In addition, GhMYB108 activated expression of *Luc* driven by the *PDF1.2, PR4*, and *PR5* promoters (Supplementary Fig. S10). These results suggest that GhMYB108 may also be able to bind to the promoters of these genes and activate their transcription.

## Discussion

### GhMYB108 and GhCML11 form a positive feedback loop to regulate *GhCML11* transcription

MYB proteins are one of the largest families of TFs and have been implicated to function in plant defense by regulating defense-related transcriptional responses (Buscaill and Rivas, 2014). In this study, we show that *GhMYB108* is a pathogen-responsive gene and that inhibition of its expression by VIGS resulted in increased disease susceptibility of cotton plants, indicating that GhMYB108 is involved in the defense response against *V*. *dahalie* in cotton. By Y2H assay, we found that GhMYB108 interacted with GhCML11 and the two proteins had a synergetic relationship. On the one hand, GhMYB108 functions as a TF to activate *GhCML11* expression. On the other hand, GhCaM11 acts as a transcriptional activator to enhance the activity of GhMYB108. Thus, the two proteins form a positive feedback loop to enhance *GhCML11* transcription. Previous work reported that the Arabidopsis MYB2 interacted with *Glycine max* CaMs for abiotic stress tolerance in Arabidopsis (Yoo *et al.*, 2005). Here, we provide a novel line of evidence showing the interaction and co-operative function of MYB and CaM, which contributed to the biotic stress tolerance in cotton.

### The *V. dahliae*-induced redistribution of Ca^2+^ may depend on the apoplastic GhCML11

Ca^2+^ plays a critical role in plant innate immunity, and Ca^2+^ influx is an early event in plant defense response against pathogen attack (Ma, 2011). In our study, we observed a clear Ca^2+^ influx into the cytosol of cotton root cells upon *V. dahliae* infection. Through comparative transcriptome analysis, we found altered expression of a number of genes encoding calcium-binding proteins. These results show that Ca^2+^ influx is tightly associated with the defense against *V. dahliae* infection in cotton.

The apoplast is a large Ca^2+^ pool in plants, and some cytosolic CaMs can be secreted into the apoplast. These apoplastic CaMs promote the entry of Ca^2+^ from the apoplast into the cytosol (Wang *et al.*, 2009; Jiang *et al.*, 2014). It was demonstrated that Ca^2+^ redistribution across the plasma membrane is required for pollen tube growth (Wang *et al.*, 2013). Using onion epidermis as an experimental system, we found that a portion of GhCML11 proteins is distributed in the apoplast. It will be interesting to investigate whether the apoplastic localization is involved in modulating the Ca^2+^ influx, which contributes to subsequent defense responses in cotton cells. In support of this notion, we found that the pathogen-induced Ca^2+^ influx was disturbed in root cells in *GhCML11*-silenced cotton plants, which was coupled with the increased disease susceptibility. It is likely that when expression of *GhCML11* was reduced, less GhCML11 protein was secreted into the apoplasts, resulting in reduced influx of Ca^2+^ into the cytosol and, as a consequence, disturbed defense responses. This result provides novel hints on the function of apoplastic CaMs in the plant immune response. Further study is required to assess the links between dynamic redistribution of Ca^2+^ and GhCML11 in defense response.

In *GhMYB108*-silenced cotton root cells, Ca^2+^ influx was also altered upon pathogen attack (Fig. 9). This could be due to reduced expression of *GhCML11*, which was caused by silencing of *GhMYB108*. In this regard, GhMYB108 is also functionally linked to the Ca^2+^ redistribution during responses to pathogen infection.

### GhMYB108, calcium, and GhCML11 function interdependently to mediate defense responses

A mechanism by which TFs, CaM, and Ca^2+^ function co-operatively to de-repress the expression of the immune system has been proposed based on studies on the Arabidopsis TF CAMTA3 (Zhang *et al.*, 2014). According to this model, plant TFs such as CAMTA3 bind to CaM and repress target gene expression prior to pathogen attack (Du *et al.*, 2009; Nie *et al.*, 2012). Upon pathogen infection, with the elevation of nuclear Ca^2+^ that binds to the CaM–TF complex, the TF is dissociated from CaM and degraded by ubiquitin-mediated destruction and, as a consequence, expression of the immune system is de-repressed (Zhang *et al.*, 2014; Fromm and Finkler, 2015). Here, we found that GhMYB108 is a transcriptional activator and GhCML11 enhances its activity in the presence of Ca^2+^. The expression of defense genes upon pathogen attack is by a mechanism of activation in this case, thus different from the mechanism involving CAMTA3.

 EMSA analysis showed that GhCML11 interacted with GhMYB108 and enhanced the transcriptional activity of GhMYB108 in a Ca^2+^-dependent manner, indicating that Ca^2+^ is critical for GhMYB108–GhCML11–DNA binding efficiency. Accordingly, transcription of *GhMYB108* or *GhCML11* in cotton roots was induced by CaCl_2_, and when cotton roots were treated with LaCl_3_ (a Ca^2+^ influx blocker), the induction of *GhMYB108* and *GhCML11* expression was inhibited upon pathogen attack (Supplementary Fig. S11). On the other hand, we found that the pathogen-responsive Ca^2+^ influx was interrupted upon silencing of *GhCML11* or *GhMYB108*, indicating that these proteins in turn had an effect on Ca^2+^ uptake which may affect the calcium-mediated signaling in the defense response. Taken together, our results demonstrate that GhMYB108, Ca^2+^, and GhCML11 can form a functional unit to regulate gene expression in cotton’s response to *V*. *dahliae* invasion.

### GhMYB108 and GhCML11 contribute to protection against *V. dahliae* invasion in cotton

Verticillium wilt is the most serious disease affecting cotton production. In our study we observed that the expression of *GhMYB108* is induced by pathogen attack and by defense-related signaling molecules; knock down of *GhMYB108* expression by VIGS impaired the disease tolerance to *V. dahliae* in cotton plants, and ectopic overexpression of *GhMYB108* enhanced disease tolerance to *V. dahliae* in transgenic Arabidopsis plants. Also, expression of a number of defense-related genes was inhibited when *GhMYB108* was silenced, and this may be the cause of the impaired disease tolerance to *V. dahliae* invasion, by either a direct or an indirect mechanism. Based on our results, we speculate that GhMYB108 is a positive regulator of the cotton defense response to *V. dahliae.*

Some CaM genes have been reported to be involved in defense responses (Yamakawa *et al.*, 2001; Park *et al.*, 2004). For example, overexpression of *GmCaM-4/-5* in wild-type Arabidopsis enhances disease resistance and induces *PR* gene expression (Park *et al.*, 2004). In this study, we found that the expression of *GhCML11* was highest in the root compared with the stem and leaves, and its expression was also induced by *V. dahliae* invasion (Supplementary Fig. S12). Cotton plants with reduced expression of *GhCML11* showed decreased disease tolerance compared with control plants (Supplementary Fig. S13). These results indicate that GhCML11 is also an important contributor in defense against Verticillium wilt in cotton. It should be mentioned that in addition to the nucleus and apoplast, GhCML11 proteins are also present in the cytoplasm. It is known that CaM in the cytosol acts as a calcium sensor and transmits the Ca^2+^ signal by interacting with target proteins (Yang and Poovaiah, 2003). Thus, apart from its roles in the nucleus and apoplast, GhCML11 may also participate in calcium signaling in the cytosol as do other CaMs.

Due to the difficulty in generating Verticillium-resistant cotton cultivars by traditional breeding, it is desirable to make breakthroughs in this field through genetic manipulation. Based on our data, we suggest that *GhMYB108* and *GhCML11* may be suitable candidate genes for molecular breeding of upland cotton cultivars with high tolerance to Verticillium wilt.

## Supplementary data

Supplementary data are available at *JXB* online.

Figure S1. Multiple sequence alignments of *GhMYB108* and potential off-target *MYB* genes.

Figure S2. Expression pattern of *GhMYB* genes upon *V. dahliae* infection in cotton plant.

Figure S3. Photobleaching phenotype in *GhCLA1*-silenced cotton plants.

Fig.ure S 4. qRT-PCR analysis of expression levels of six potential off-target *MYB* genes in control and *GhMYB108*-silenced plants.

Figure S5. Disease symptoms of *GhMYB108*-overexpressing Arabidopsis plants inoculated with *B. cinerea* or *Pst* DC3000.

Figure S6. The Ca^2+^-dependent mobility shift assay of GhCML11.

Figure S 7. Expression of *PDF1.2*, *PR4*, and *PR5* genes in *GhMYB108*-silenced and *GhMYB108*-overexpressing plants, and binding of GhMYB108 to their promoter sequences.

Figure S8. Verification of transcriptomic data in *GhMYB108*-silenced cotton plants.

Figure S9. Expression levels of defense-related genes in *GhMYB108* transgenic Arabidopsis plants.

Figure S10. Transient expression analysis of GhCML11-enhanced transcriptional activation activity of GhMYB108.

Figure S11. Effects of Ca^2+^ on the expression of *GhMYB108* and *GhCML11.*

Figure S12. Expression pattern of *GhCML11* in cotton plants.

Figure S13. Increased susceptibility of *GhCML11*-silenced cotton plants to *V. dahliae*.

Table S1. Primers used in this study.

Table S2. Genes differentially expressed in *GhMYB108*-silenced plants determined by comparative transcript profiling analysis.

Table S3. Defense-related genes down-regulated in *GhMYB108*-silenced cotton plants.

Supplementary Data
