# Supplementary material for: The cotton MYB108 forms a positive feedback regulation loop with CML11 and participates in the defense response against Verticillium dahliae infection
Source: J Exp Bot. 2016 Feb 11;67(6):1935–50. doi: 10.1093/jxb/erw016 (PMC4783372; doi:10.1093/jxb/erw016)
Supplement: Supplementary Data [file supp_67_6_1935__index.html]

The cotton MYB108 forms a positive feedback regulation loop with CML11 and participates in the defense response against Verticillium dahliae infection — The cotton MYB108 forms a positive feedback regulation loop with CML11 and participates in the defense response against Verticillium dahliae infection — Supplementary Data 

# The cotton MYB108 forms a positive feedback regulation loop with CML11 and participates in the defense response against *Verticillium dahliae* infection

## Supplementary Data

Data files

- supplementary\_figures\_S1\_S13\_Table\_S1\_\_S3.pdf - Supplementary Data
- supplementary\_table\_S2.xls - Supplementary Data
