# Supplementary material for: The cotton MYB108 forms a positive feedback regulation loop with CML11 and participates in the defense response against Verticillium dahliae infection
Source: J Exp Bot. 2016 Feb 11;67(6):1935–50. doi: 10.1093/jxb/erw016 (PMC4783372; doi:10.1093/jxb/erw016)
Supplement: Supplementary Data [file supp_erw016_supplementary_figures_S1_S13_Table_S1__S3.pdf]

**Supplementary material**

# The cotton MYB108 forms a positive feedback regulation loop with CML11 and participates in defense response against *Verticillium dahliae* infection

**Huan-Qing Cheng, Li-Bo Han, Chun-Lin Yang, Xiao-Min Wu, Nai-Qin Zhong, Jia-He Wu, Fu-Xin Wang, Hai-Yun Wang* and Gui-Xian Xia***

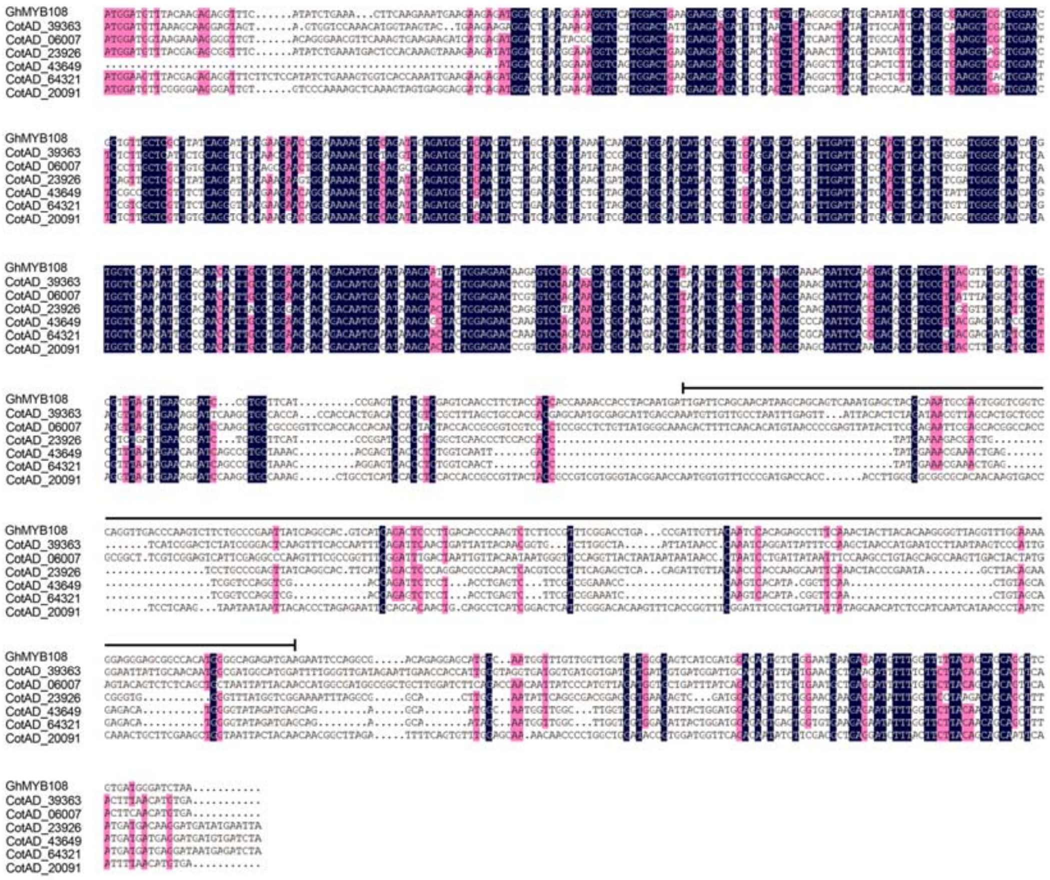

**Supplementary Fig. S1.** Multiple sequence alignments of *GhMYB108* and potential off-target *MYB* genes.

Sequence used for *GhMYB108* VIGS construct was marked with single line. CotAD_39363, CotAD_06007, CotAD_23926, CotAD_43649, CotAD_64321, CotAD_20091: potential off-target *MYB* genes.

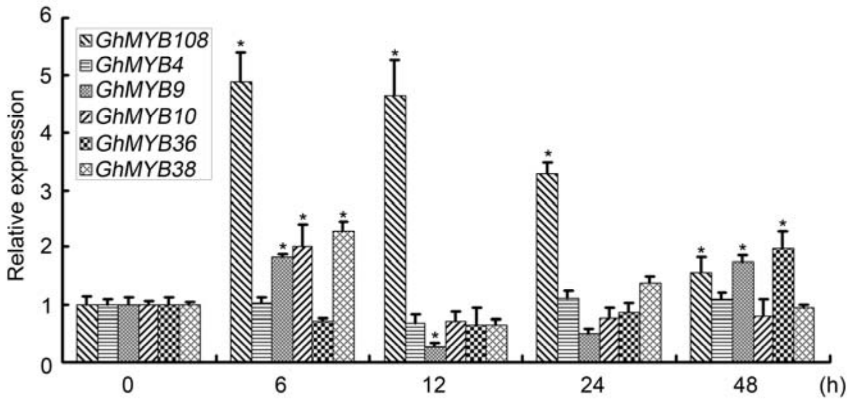


**Supplementary Fig. S2.** Expression pattern of *GhMYB* genes upon *V. dahliae* infection in cotton plant.

Expression of *GhMYB108*, *GhMYB4*, *GhMYB9*, *GhMYB10*, *GhMYB36*, and *GhMYB38* genes in cotton roots in response to *V. dahliae* infection. Error bars represent the SD of three biological replicates. Asterisks indicate statistically significant differences, as determined by Student's *t*-test (*$P < 0.05$).

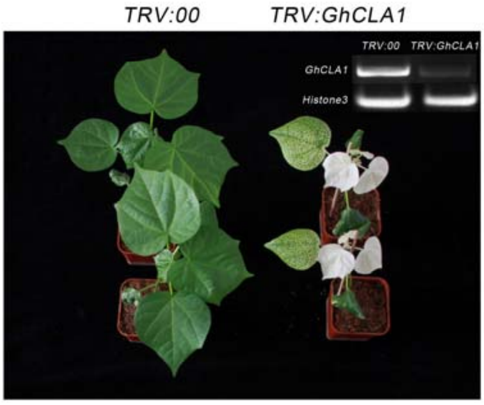


**Supplementary Fig. S3.** Photobleaching phenotype in *GhCLA1*-silenced cotton plants.

The *GhCLA1* gene was used as a positive control with a photobleaching phenotype after VIGS in cotton. Plants infiltrated with *Agrobacterium* containing empty vector (*TRV:00*) or pTRV2-GhCLA1 (*TRV2:GhCLA1*) showed different phenotypes in leaves. Photo was taken 14 days after VIGS. The inset indicated the expression levels of *GhCLA1* in control and *GhCLA1*-silenced cotton plants analyzed by RT-PCR. *Histone3* was used as an internal control.

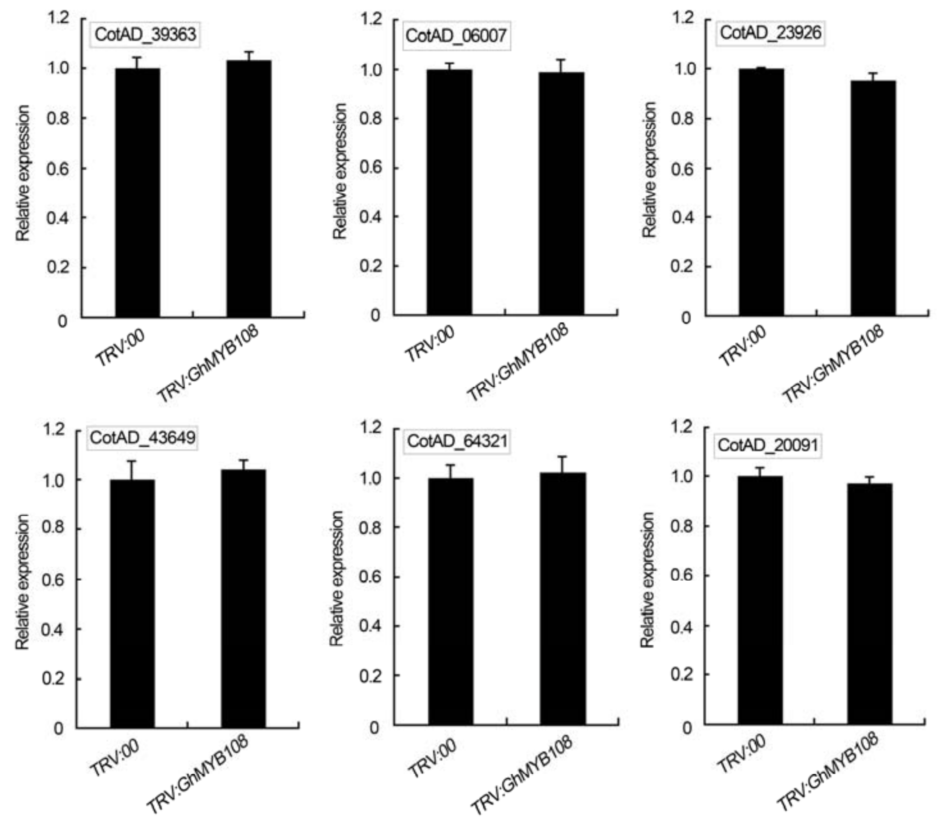


**Supplementary Fig. S4.** qRT-PCR analysis of expression levels of six potential off-target *MYB* genes in control and *GhMYB108*-silenced plants.

*TRV:00*: control plants; *TRV:GhMYB108*: *GhMYB108*-silenced plants. Error bars represent the SD of three biological replicates.

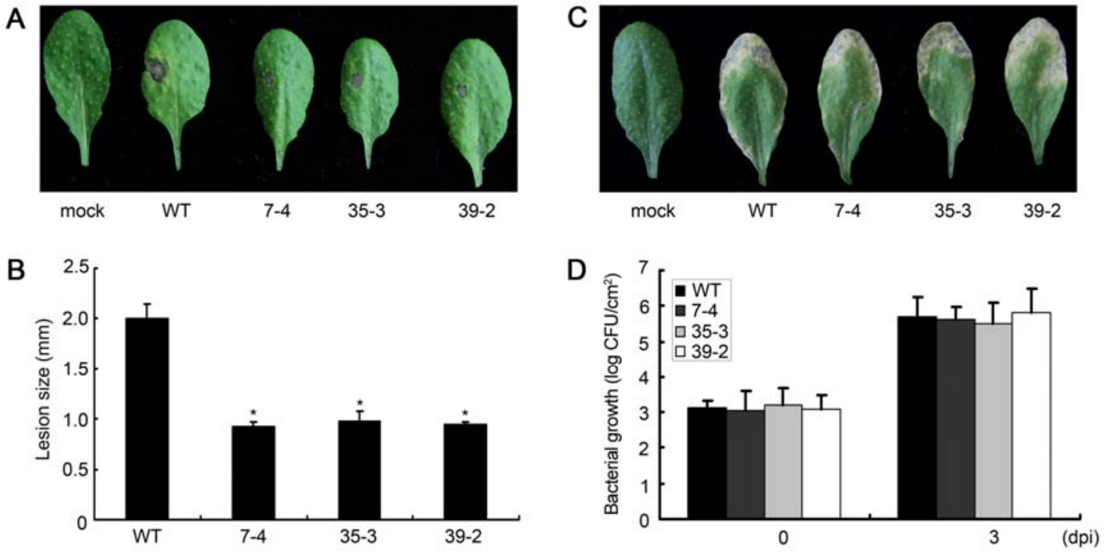


**Supplementary Fig. S5.** Disease symptoms of *GhMYB108*-overexpressing Arabidopsis plants inoculated with *B. cinerea* or *Pst* DC3000.

(A) Photographs of leaves 3 days post inoculation with *B. cinerea* between WT (wild type) and *GhMYB108* transgenic Arabidopsis lines (7-4, 35-3 and 39-2). (B) Lesion size measured at 3 days after *B. cinerea* inoculation. Error bars represent the SD (n = 25) of three biological replicates. Asterisks indicate statistically significant differences, as determined by Student's *t*-test (*$P < 0.05$). (C) Symptoms of WT and *GhMYB108* transgenic Arabidopsis lines infected with *Pst* DC3000. Photos were taken 3 days post inoculation. (D) Growth of *Pst* DC3000 in infected leaves of WT and *GhMYB108* transgenic plants. Error bars represent the SD (n = 20) of three biological replicates**.**

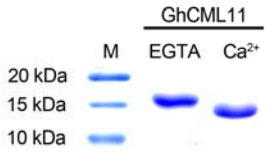


**Supplementary Fig. S6.** The $Ca^{2+}$-dependent mobility shift assay of GhCML11. GhCML11 proteins were separated on a 15% SDS-PAGE gel in the presence of $Ca^{2+}$ or EGTA in sample buffers. Protein bands were visualized with Coomassie brilliant blue staining. M: molecular weight marker (kDa).

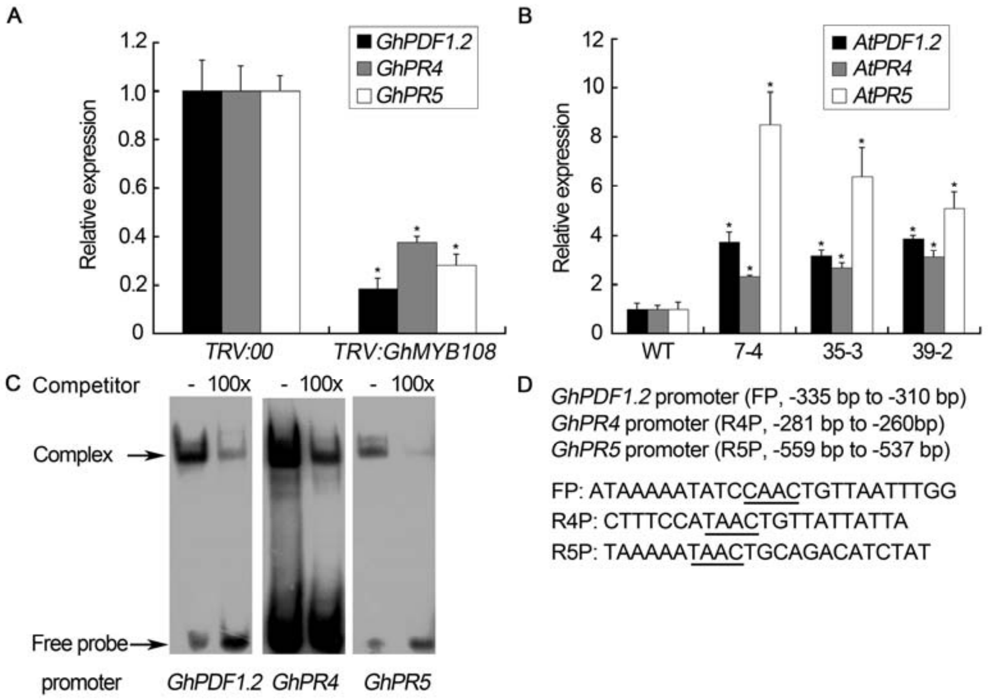


**Supplementary Fig. S7.** Expression of *PDF1.2*, *PR4*, and *PR5* genes in *GhMYB108*-silenced and *GhMYB108*-overexpressing plants and binding of GhMYB108 to their promoter sequences.

(A) Expression levels of *GhPDF1.2*, *GhPR4*, and *GhPR5* in control (*TRV:00*) and GhMYB108-silenced (*TRV:GhMYB108*) cotton plants. (B) Expression levels of *AtPDF1.2*, *AtPR4*, and *AtPR5* in WT (wild-type) and *GhMYB108* transgenic Arabidopsis plants (7-4, 35-3, and 39-2). Asterisks indicate statistically significant differences, as determined by Student's *t*-test ($*P < 0.05$). (C) Binding of GhMYB108 to the promoter sequences of *GhPDF1.2*, *GhPR4*, and *GhPR5*. EMSA analysis was performed with the purified recombinant GhMYB108 proteins and DNA sequences containing the promoter region of *GhPDF1.2*, *GhPR4*, and *GhPR5* genes, respectively. Purified proteins were incubated with labeled DNA probes from corresponding genes in the absence or presence of 100-fold molar excess of unlabelled probes. (D) The promoter sequences used in EMSA. Numbers indicate positions relative to the translation start site of the corresponding gene. The underlined sequence indicates the core motif of MYB binding site.

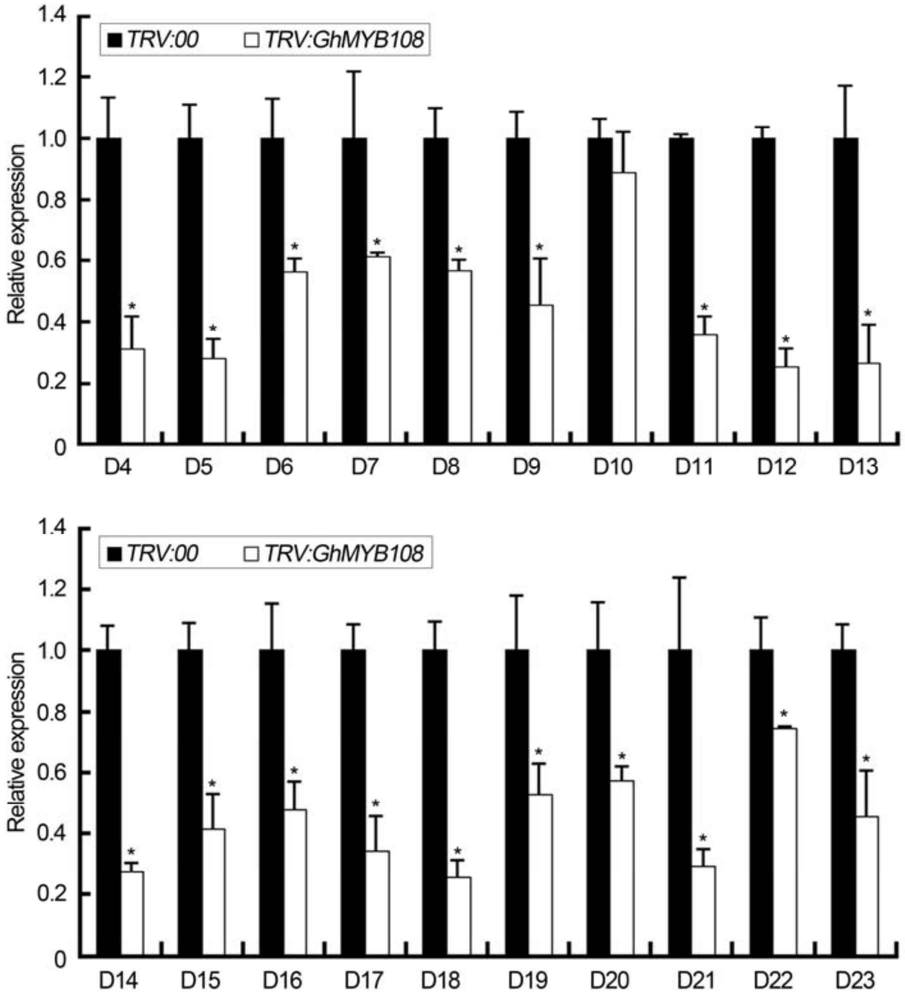


**Supplementary Fig. S8.** Verification of transcriptomic data in *GhMYB108*-silenced cotton plants.

The down-regulated defense-related genes from the transcriptomic data were verified by qRT-PCR. *TRV:00*: control plants; *TRV:GhMYB108*: *GhMYB108*-silenced plants. Error bars represent the SD of three biological replicates. Asterisks indicate significant differences, as determined by Student's *t*-test (**P* < 0.05).

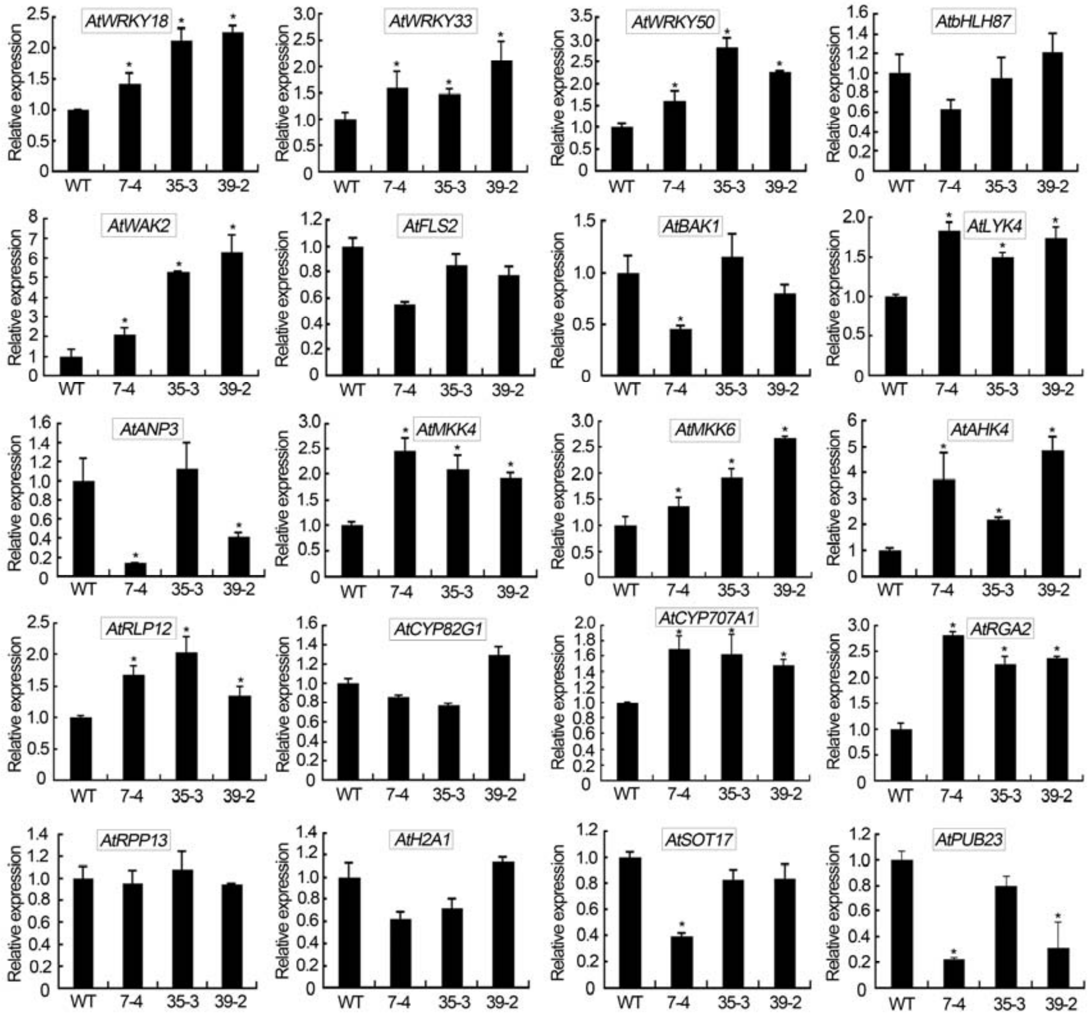


**Supplementary Fig. S9.** Expression levels of defense-related genes in *GhMYB108* transgenic Arabidopsis plants.

qRT-PCR analysis of expression levels of defense-related genes in *GhMYB108* transgenic Arabidopsis plants. WT: wild-type plants; 7-4, 35-3, 39-2: *GhMYB108* transgenic lines. Error bars represent the SD of three biological replicates. Asterisks indicate statistically significant differences, as determined by Student's *t*-test (*$P < 0.05$).

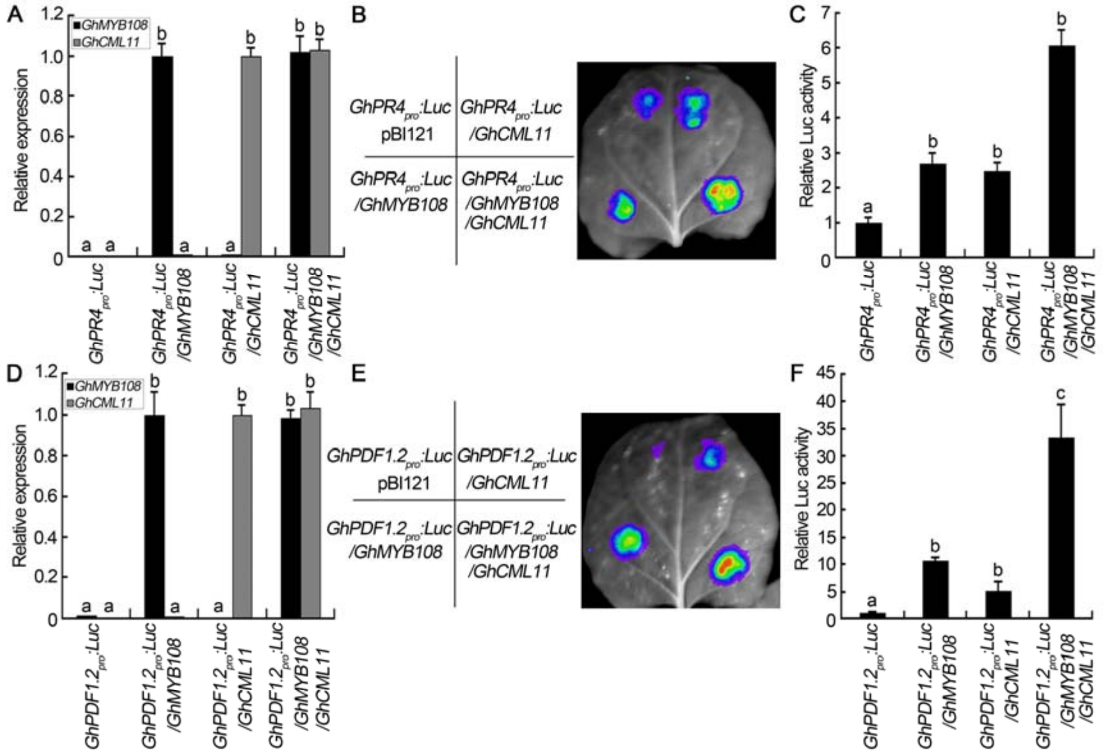


**Supplementary Fig. S10.** Transient expression analysis of GhCML11-enhanced transcriptional activation activity of GhMYB108.

(A and D) Expression levels of *GhMYB108* and *GhCML11* in tobacco leaves transformed with indicated constructs in (B) and (E). Different letters indicate significant differences at $P < 0.01$ (Student's *t*-test, $n \geq 15$, 3 biological repeats). (B and E) Luminescence signal on *N. benthamiana* leaves. Luminescence imaging was performed 48 h after co-infiltrated with *Agrobacterium* strains containing indicated constructs on the left panel. (C and F) Quantitative analysis of luminescence intensity in (B) and (E). Different letters indicate significant differences at $P < 0.05$ (Student's *t*-test, $n = 30$, 3 biological repeats).

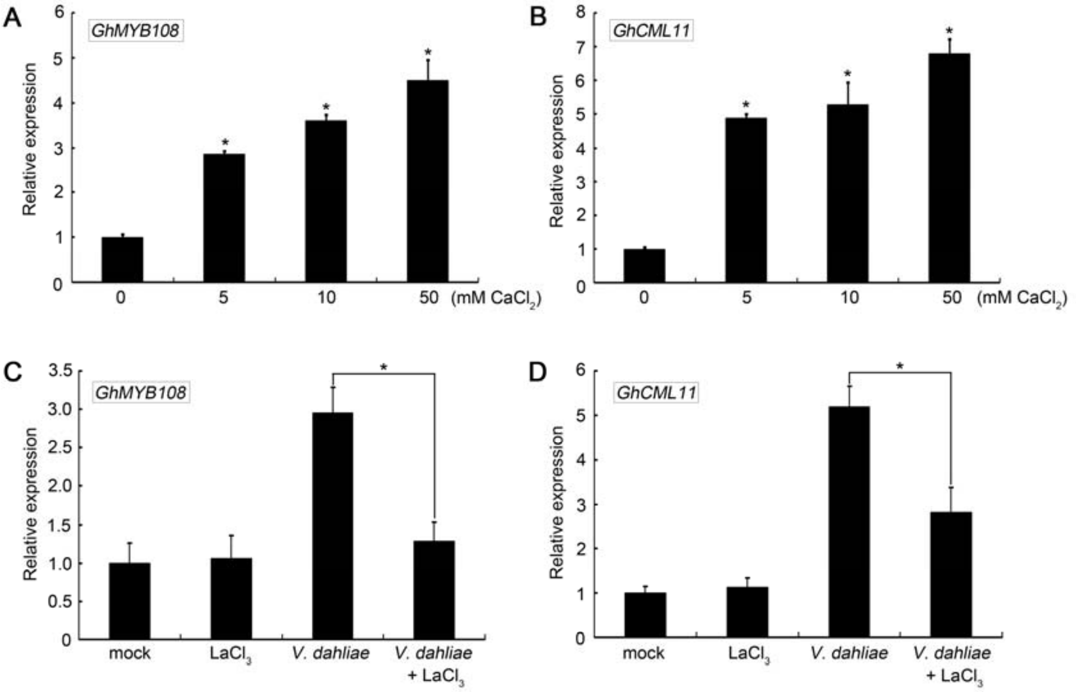


**Supplementary Fig. S11.** Effects of $Ca^{2+}$ on the expression of *GhMYB108* and *GhCML11*.

(A and B) qRT-PCR analysis of expression levels of *GhMYB108* and *GhCML11* after treatment of cotton roots with different concentrations of $CaCl_2$. (C and D) qRT-PCR analysis of expression levels of *GhMYB108* and *GhCML11* after treatment with $LaCl_3$ before and after *V. dahliae* inoculation. $LaCl_3$, $Ca^{2+}$ influx blocker. Error bars represent the SD of three biological replicates. Asterisks indicate statistically significant differences, as determined by Student's *t*-test (*$P < 0.05$).

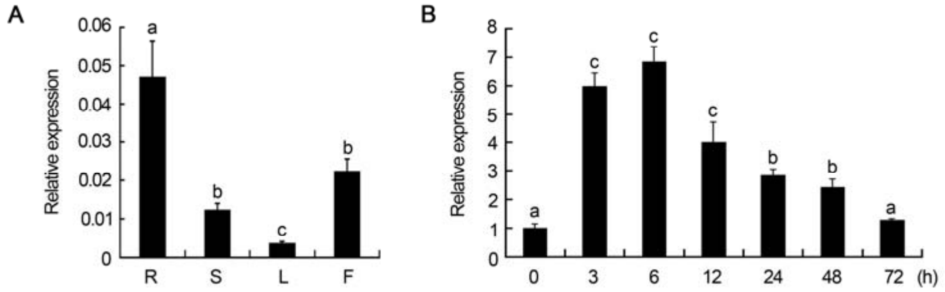


**Supplementary Fig. S12.** Expression pattern of *GhCML11* in cotton plants.

(A) qRT-PCR analysis of *GhCML11* gene expression in root (R), stem (S), leaf (L), and flower (F) of cotton plants. Error bars represent the SD of three biological replicates. (B) Accumulation of *GhCML11* transcripts in cotton roots in response to *V. dahliae* infection. Error bars represent the SD of three biological replicates. Different letters indicate significant differences, as determined by Student's *t*-test ($P < 0.05$).

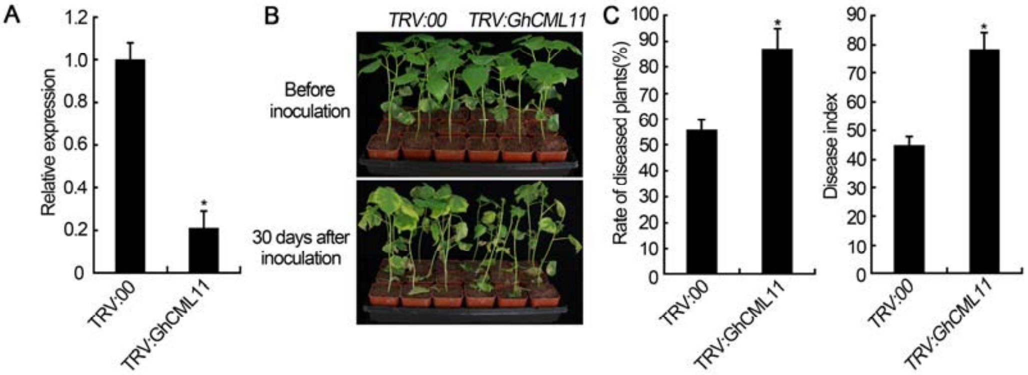


**Supplementary Fig. S13.** Increased susceptibility of *GhCML11*-silenced cotton plants to *V. dahliae*.

(A) Analysis of *GhCML11* expression levels. Total RNAs were extracted from leaves of cotton plants at 14 days post agroinfiltration and the expression level of *GhCML11* in VIGS plants was compared with that of control plants (*TRV:00*). Error bars represent the SD of three biological replicates. Asterisks indicate statistically significant differences, as determined by Student's *t*-test (*$P$ < 0.05). (B) Disease symptom of control (*TRV:00*) and *GhCML11*-silenced (*TRV:GhCML11*) plants infected by *V. dahliae*. (C) Rate of diseased plants and disease index of the control and *GhCML11*-silenced plants. Error bars represent the SD of three biological replicates (n ≥ 30). Asterisks indicate statistically significant differences, as determined by Student's *t*-test (*$P$ < 0.05).

**Supplementary Table S1.** Primers used in this study.

| Primer name | Sequence (5' to 3') |
|---|---|
| GhMYB4-qRT-F | AAGCGAGGAGAGAGAGAGCCTGGTG |
| GhMYB4-qRT-R | TTCGGTGAAAATGTTTTCTTCAATC |
| GhMYB9-qRT-F | ATTACCAGCAAACCCAACCAGAGTC |
| GhMYB9-qRT-R | ATTCCAAGATACCAGATTTCAAGCC |
| GhMYB10-qRT-F | TGATTCGTCACCCTCACCGGATCAG |
| GhMYB10-qRT-R | AGAAGAAGAGCAGAATTATTGATGG |
| GhMYB36-qRT-F | TCCTATGCAGCAGCACTTTCCGTTG |
| GhMYB36-qRT-R | CAGTACTGATATTCGTATCATGGCC |
| GhMYB38-qRT-F | ACGGGGAGTACAGTCATGGTACCGA |
| GhMYB38-qRT-R | TAATACAACAAAGTGCGAACTTTGG |
| GhMYB108-qRT-F | ACGTCATCAGACTCCCTTGACACCC |
| GhMYB108-qRT-R | ACCAACAAACCATTGCCATGCTCCT |
| GhMYB108-pBI121-F | CGGCTCTAGAATGGATGTTTACAAG |
| GhMYB108-pBI121-R | CCAGAGCTCGATCCCATCACGAAGC |
| GhMYB108-5-race-R | TCAACTAAACGGGGGATCCAAACGTAACGC |
| GhMYB108-3-race-F | TCAACCTTCTACCACCACCAAAACCACCTA |
| GhMYB108-mbp-F | CTAGTCGACATGGATGTTTACAAGAGAGG |
| GhMYB108-mbp-R | GACAAGCTTTTAGATCCCATCACGAAGC |
| GhMYB108-gfp-F | CTAGTCGACATGGATGTTTACAAGAGAGG |
| GhMYB108-gfp-R | CTTGGTACCGATCCCATCACGAAGCTGC |
| GhMYB108ΔC-gfp-F | ACTCGTCGACATGGATGTTTACAAGAGAG |
| GhMYB108ΔC-gfp-R | CTACAAGCTTTTAAACTAAACGGGGGATC |
| GhMYB108ΔN-gfp-F | ACTCGTCGACATGAACGGATCCGTGCTTC |
| GhMYB108ΔN-gfp-R | CATCAAGCTTTTAGATCCCATCACGAAGC |
| GhMYB108-vigs-F | CGACGAATTCTTGATTCAGCAAC |
| GhMYB108-vigs -R | ACTAGGTACCTTCATCTCTGCCC |
| GhMYB108-dlr-F | CGGCTCTAGAATGGATGTTTACAAGAGAGG |
| GhMYB108-dlr-R | CGATGTCGACGATCCCATCACGAAGCTGC |
| GhMYB108-BD-F | CGACCATATGATGGATGTTTACAAGAGAG |
| GhMYB108-BD-R | CTCAGTCGACTTAGATCCCATCACGAAGC |
| GhMYB108-AD-F | CGACCATATGATGGATGTTTACAAGAGAG |
| GhMYB108-AD-R | CTTCGGATCCTTAGATCCCATCACGAAGC |
| GhCML11-AD-F | CGACCATATGATGGGTGATATACTAACTC |
| GhCML11-AD-R | CATAGGATCCTCATCCAACGGTCGTCATC |
| GhCML11-BD-F | CGACCATATGATGGGTGATATACTAACTC |
| GhCML11-BD-R | CTACGTCGACTCATCCAACGGTCGTCATC |
| GhMYB108-NLuc-F | CCGAGGTACCATGGATGTTTACAAGAGAG |
| GhMYB108-NLuc-R | CATGGTCGACGATCCCATCACGAAGCTGC |
| GhMYB108-pro-R1 | GTGGATATTGACATGCGCCTTAAGC |
| GhMYB108-pro-R2 | CATCCATTTCTCAACTTAAAACAGG |
| ITS1-F | AAAGTTTTAATGGTTCGCTAAGA |
| ST-VE1-R | CTTGGTCATTTAGAGGAAGTAA |

| | |
|---|---|
| GhCML11-CLuc-R | CATGGTCGACTCATCCAACGGTCGTCATC |
| GhCML11-mCherry-F | CGACGGATCCATGGGTGATATACTAACTC |
| GhCML11-mCherry-R | CATCGTCGACTCCAACGGTCGTCATCATT |
| GhCML11-gst-F | CATAGGATCCATGGCGGACCAGCTTACCG |
| GhCML11-gst-R | CGCCGTCGACTTAAAGAAAAATGCATTTACG |
| GhCML11-vigs-F | CTACGGATCCCCAAGGTACCAAATTGA |
| GhCML11-vigs-R | CATAGGTACCACTTTGGGAAGTTTAAG |
| GhCML11-qRT-F | ATTTCCTTGCATTGAAACTCC |
| GhCML11-qRT-R | GTTTTGTTTGTTTTGGTGAGC |
| $GhCML11_{pro}$-pGWB435-F | TCCAGCTCACCAAAACAAACAAAAC |
| $GhCML11_{pro}$-pGWB435-R | CGAGGGTCTCAAGAAGGTACAGCCT |
| $GhPDF1.2_{pro}$-pGWB435-F | ATTCCTCGAAAAATAATAATCTAGAAAGA |
| $GhPDF1.2_{pro}$-pGWB435-R | TAAATATTATTATAACCGAGCGAATGATTGAAA |
| $GhPR4_{pro}$-pGWB435-F | TTTGGGGGTCCAAAATGAAGCCACC |
| $GhPR4_{pro}$-pGWB435-R | TTCAGTTTTGTGTAATTTTCATGCT |
| $GhPR5_{pro}$-pGWB435-F | ATCAAATATAATCCTTATTAATAATTCAAT |
| $GhPR5_{pro}$-pGWB435-R | GGTTGGTTTTTACTTGGTGATTTGTTCATA |
| GhPR4-qRT-F | GCTACCTACCATTACTACAACCCTG |
| GhPR4-qRT-R | TTGCTGCACTGATCCACAATTCTTA |
| GhPR5-qRT-F | AGCCGCCTCAGCGTTTATTTTTAAA |
| GhPR5-qRT-R | TACGAGCCATGGCTGTGCCAGCAG |
| GhPDF1.2-qRT-F | CTGTGGTAGCGGATGGTGATAAG |
| GhPDF1.2-qRT-R | GTGCAGACGCATTTGCGAAGGAA |
| Histone3-F | GCCAAGCGTGTCACAATTATGC |
| Histone3-R | ACATCACATTGAACCTACCACTACC |
| AtPR4-qRT-F | GAAGAACACAAGAACAAATGCTGCA |
| AtPR4-qRT-R | GTAGACCGATCGATATTGACCTCAA |
| AtPR5-qRT-F | GCTACGCTTATGACGACGAAACGAG |
| AtPR5-qRT-R | AAATCAGCTGAGTGTAACAACTGAC |
| AtPDF1.2-qRT-F | GGAGCCAAACATGGATCATGCAACT |
| AtPDF1.2-qRT-R | TGTAACAACAACGGGAAAATAAACA |
| AtEF-1α-F | TTGGCGGCACCCTTAGCTGGATCA |
| AtEF-1α -R | ATGCCCCAGGACATCGTGATTTCAT |
| GhEHD2-qRT-F | GGCAAAGCTAAAGCTCAAC |
| GhEHD2-qRT-R | CTGGGATTTCATATCCTAAC |
| GhPBP1-qRT-F | TTGGAAACCTCAACTCACC |
| GhPBP1-qRT-R | TGATGGAGGAATCGCAGAC |
| GhNRT1.2-qRT-F | ACCTTACCCGTGACTTTCC |
| GhNRT1.2-qRT-R | GTGCATCGCCTTCCTCGTA |
| GhRBOHF-qRT-F | AGCAGCAATGAATCAACCA |
| GhRBOHF-qRT-R | GTGCATCGCCTTCCTCGTA |
| GhIQD1-qRT-F | ATACAATGCCGACTTCAGA |
| GhIQD1-qRT-R | GTGGATACAGTGGCCGAGA |
| GhIQD14-qRT-F | AAGGCATGGAGAAACCAAT |

| | |
|---|---|
| GhIQD14-qRT-R | AAGCATCTTTATGAGGCAC |
| GhIQD31-qRT-F | TGATTTGGCTGTGGCTCCTC |
| GhIQD31-qRT-R | CGCCGATTGTTCTTGCTTTAT |
| GhCLA1-vigs-F | GCTCGAATTCCACAACATCGATGAT |
| GhCLA1-vigs-R | CCGAGAGCTCATGATGAGTAGATTGCACAA |
| GhCIPK6-qRT-F | AAAACCCATCTTTACTCCAT |
| GhCIPK6-qRT-R | TGATCTGCTCCATCATCCC |
| CotAD_39363-qRT-F | CGGGACTCAAGTTTCACCAAT |
| CotAD_39363- qRT -R | CATCACCATCACCTACCCAAT |
| CotAD_06007- qRT -F | CAATAATGGGTTCCAGGTTAC |
| CotAD_06007- qRT -R | ACATGGGATAATTGTTGGTCT |
| CotAD_23926- qRT -F | TACCCGAATAGCTTACAGAAC |
| CotAD_23926- qRT -R | CATATCATCCTTGTCATCATAAA |
| CotAD_43649- qRT -F | CTTCGTCGGAAACCCAAGTCA |
| CotAD_43649- qRT -R | AAGCTGCTGTTGTAAGAACCAA |
| CotAD_64321- qRT -F | CTTCGTCGGAAATCCAAGTCA |
| CotAD_64321- qRT -R | AAGCTGCTGTTGTAAGAACCA |
| CotAD_20091- qRT -F | TCATAACCCTAATCCAAACTG |
| CotAD_20091- qRT -R | TTGCTGCTGTAAGAAGTAAA |
| D4-qRT-F | CACAACCATCTTCCTCCTT |
| D4-qRT-R | GTAGCCATCTGTTCCACCA |
| D5-qRT-F | AGTCAGCCTAACAATACAATG |
| D5-qRT-R | AGACAGTAATCCGTCCAAA |
| D6-qRT-F | TATGGGAAGAAATGGGTGAA |
| D6-qRT-R | GCGAGATAGTAACGGTAAAA |
| D7-qRT-F | CCAGAGGCCATTGCACAAA |
| D7-qRT-R | TGCTCCCACCAGGAACCAG |
| D8-qRT-F | TTGACCCTGAGTATTTCCA |
| D8-qRT-R | GCCATTGCCGTTACCATTT |
| D9-qRT-F | GTGGCTAATCATCACTGCA |
| D9-qRT-R | GTGGGAATAGACCCGACAG |
| D10-qRT-F | GCTAATCCAGGTGGCTCTG |
| D10-qRT-R | TGGGCTTGGTTGAACTCTT |
| D11-qRT-F | AGCACTTGATTGAACCCTC |
| D11-qRT-R | ATCCCATGTCGATGAACTA |
| D12-qRT-F | CCGAGACGACGACACCCAT |
| D12-qRT-R | CGAACCGATCCAACAAGCT |
| D13-qRT-F | GTAGGCTGGCGGAACTAAC |
| D13-qRT-R | CATAAAGGCGAGACGAAGG |
| D14-qRT-F | ATGTGCTATTGGACGTTTC |
| D14-qRT-R | ATGCTCTTTCTTTAGGGTT |
| D15-qRT-F | CAGGCAAATGAACAGATGA |
| D15-qRT-R | TGCTACAGCCAGGTAAAGA |
| D16-qRT-F | GTTTATGTTTGGGCTGCTA |

| | |
|---|---|
| D16-qRT-R | GAAATGGTGCCTGTGAGAT |
| D17-qRT-F | AATACCTCCCATAATCACCA |
| D17-qRT-R | ATTGGCTCTTTCCCACCTA |
| D18-qRT-F | TTTGATCCCTCAAGATTTG |
| D18-qRT-R | GCTGTTACTACCCACCATT |
| D19-qRT-F | CACTTCACTTATTTCGTCTACC |
| D19-qRT-R | ATTCTACTCTTGCCCACCA |
| D20-qRT-F | CTGGAAAGACTACCAAACC |
| D20-qRT-R | CGATGTATCACCAAATGCT |
| D21-qRT-F | CCCTCACATTCACAAGTCT |
| D21-qRT-R | ATCCCTACCCTAAACAAAG |
| D22-qRT-F | TCATCGGTTATCCTTTCTC |
| D22-qRT-R | AGCAGCCATTTCAGGTGTC |
| D23-qRT-F | TGCGAGATGATACTAACGG |
| AtWRKY18-qRT-F | TAGCGACATACGAAGGGAC |
| AtWRKY18-qRT-R | ATAGCAGCAGCAAGAGCAG |
| AtWRKY33-qRT-F | TGCTATTGCTGGTCACTCC |
| AtWRKY33-qRT-R | AGGTCTCCTCGTTTGGTTC |
| AtWRKY50-qRT-F | GTATGGGAAGAAGATGGTG |
| AtWRKY50-qRT-R | CTTGAGTGATTGTGGGAAC |
| AtbHLH87-qRT-F | ACTTCTGTGACAAGGGTGG |
| AtbHLH87-qRT-R | GCAATTATCTTACGCTATCT |
| AtWAK2-qRT-F | CCGACAATGACCGAAGTAG |
| AtWAK2-qRT-R | GTTTCTGAGGGATAACGAC |
| AtFLS2-qRT-F | AACGACCCTTTAGGAGTATTA |
| AtFLS2-qRT-R | TATGGCTGGAGACAGAACA |
| AtBAK1-qRT-F | CTGGGTGAAAGGGTTGTTA |
| AtBAK1-qRT-R | GTTGGGTAGTTGAAATCTTGTCT |
| AtLYK4-qRT-F | GAGTTGCGGTTCTTGAGCT |
| AtLYK4-qRT-R | ACAGATGGACGCGAGTTAA |
| AtANP3-qRT-F | AGATTAACTCTAGTATCCGTAGC |
| AtANP3-qRT-R | GACCCTGAGTCTTCTCCTT |
| AtMKK4-qRT-F | GTCGCCGTCCTGATCTTAC |
| AtMKK4-qRT-R | ATACCGTTCCACCTGCTCC |
| AtMKK6-qRT-F | CTTGGCAGCAATAGTAGAG |
| AtMKK6-qRT-R | AACAGGTGGTTCCAGAGTG |
| AtAHK4-qRT-F | GATGGACGGATTTGAAGCA |
| AtAHK4-qRT-R | TACGACGAAGGTGAGATAGGA |
| AtRLP12-qRT-F | CTCCCTCCTTGTTCACTCT |
| AtRLP12-qRT-R | TGACTCCATTCCAAAGACA |
| AtCYP82G1-qRT-F | GCCTCTATTCGGACACCTC |
| AtCYP82G1-qRT-R | TGCTGTAGCCAAGTCGTTG |
| AtCYP707A1-qRT-F | TTCATCATCTCACCACCAA |
| AtCYP707A1-qRT-R | AAGGCAATTCTGTCATTCTAC |

| | |
|---|---|
| AtRGA2-qRT-F | TGCTATTGCTGGTCACTCC |
| AtRGA2-qRT-R | GAGGAGCATTCAGCTACGA |
| AtRPP13-qRT-F | GGATAGTAGAAGAAGGGTCG |
| AtRPP13-qRT-R | AAATTGAACATCGGGAATG |
| AtH2A1-qRT-F | AGTTGGCGATTAGAGGAGA |
| AtH2A1-qRT-R | GGATAAAGGAAAATGAGTC |
| AtSOT17-qRT-F | ACTGGCTTTCTTTCTTCAA |
| AtSOT17-qRT-R | GTTCACCACATATTCCTCC |
| AtPUB23-qRT-F | AAGCGATAGAGCGGTTAGG |
| AtPUB23-qRT-R | TCAGCAGGGATATGCAAGA |

**Supplementary Table S3.** Defense-related genes down-regulated in *GhMYB108*-silenced cotton plants.

| Custom ID | Gene ID | Ratio | Annotation |
|---|---|---|---|
| D1 | Unigene24024 | -3.219552634 | Defensin-like protein 16 (PDF1.2) |
| D2 | Unigene39636 | -2.144169535 | Pathogenesis-related protein PR-4A |
| D3 | Unigene24561 | -1.109232546 | Pathogenesis-related protein 5 |
| D4 | CL11789 | -1.392639921 | WRKY transcription factor 18 |
| D5 | CL5469 | -1.103135385 | Probable WRKY transcription factor 33 |
| D6 | Unigene14461 | -1.22437439 | Probable WRKY transcription factor 50 |
| D7 | CL4272 | -1.496137059 | Transcription factor bHLH87 |
| D8 | CL3156 | -2.951792885 | Wall-associated receptor kinase 2 |
| D9 | CL5714 | -1.660371486 | LRR receptor-like serine/threonine-protein kinase FLS2 |
| D10 | Unigene13686 | -1.236825379 | BRASSINOSTEROID INSENSITIVE 1-associated receptor kinase 1(BAK1) |
| D11 | Unigene11352 | -1.017808689 | LysM domain receptor-like kinase 4 |
| D12 | CL10768 | -1.206741829 | Mitogen-activated protein kinase kinase kinase 3 |
| D13 | CL6810 | -1.25759391 | Mitogen-activated protein kinase kinase 4 |
| D14 | Unigene3343 | -1.257265737 | Mitogen-activated protein kinase kinase 6 |
| D15 | Unigene42675 | -1.363014048 | Histidine kinase 4 |
| D16 | CL2475 | -6.764145213 | Receptor-like protein 12 |
| D17 | CL9316 | -2.240225367 | Cytochrome P450 82G1 |
| D18 | CL4312 | -2.189863033 | Abscisic acid 8'-hydroxylase1 (CYP707A1) |
| D19 | Unigene27837 | -2.533284106 | Disease resistance protein RGA2 |
| D20 | Unigene11468 | -1.469416175 | Disease resistance protein RPP13 |
| D21 | Unigene14161 | -1.807599783 | Histone H2A variant 1 |
| D22 | Unigene15182 | -1.668735646 | Cytosolic sulfotransferase 17 |
| D23 | CL1628 | -1.009230557 | E3 ubiquitin-protein ligase PUB23 |
